# Comparisons of Transcriptional Profiles of Gut Genes between Cry1Ab-Resistant and Susceptible Strains of *Ostrinia nubilalis* Revealed Genes Possibly Related to the Adaptation of Resistant Larvae to Transgenic Cry1Ab Corn

**DOI:** 10.3390/ijms18020301

**Published:** 2017-01-30

**Authors:** Jianxiu Yao, Yu-Cheng Zhu, Nanyan Lu, Lawrent L. Buschman, Kun Yan Zhu

**Affiliations:** 1 Department of Entomology, 123 Waters Hall, Kansas State University, Manhattan, KS 66506, USA; jianxiu.yao@ars.usda.gov (J.Y.); lbuschma@ksu.edu (L.L.B.); 2 U.S. Department of Agriculture-Agricultural Research Service, 141 Experiment Station Rd, Stoneville, MS 38776, USA; yc.zhu@ars.usda.gov; 3 Bioinformatics Center, Kansas State University, Manhattan, KS 66506, USA; nanyan@ksu.edu; 4 963 Burland Drive, Bailey, CO 80421, USA

**Keywords:** *Bacillus thuringiensis*, Cry1Ab, microarray, European corn borer, gene expression, *Ostrinia nubilalis*, transgenic corn

## Abstract

A microarray developed on the basis of 2895 unique transcripts from larval gut was used to compare gut gene expression profiles between a laboratory-selected Cry1Ab-resistant (R) strain and its isoline susceptible (S) strain of the European corn borer (*Ostrinia nubilalis*) after the larvae were fed the leaves of transgenic corn (MON810) expressing Cry1Ab or its non-transgenic isoline for 6 h. We revealed 398 gut genes differentially expressed (i.e., either up- or down-regulated genes with expression ratio ≥2.0) in S-strain, but only 264 gut genes differentially expressed in R-strain after being fed transgenic corn leaves. Although the percentages of down-regulated genes among the total number of differentially expressed genes (50% in S-strain and 45% in R-strain) were similar between the R- and S-strains, the expression ratios of down-regulated genes were much higher in S-strain than in R-strain. We revealed that 17 and 9 significantly up- or down-regulated gut genes from S and R-strain, respectively, including serine proteases and aminopeptidases. These genes may be associated with Cry1Ab toxicity by degradation, binding, and cellular defense. Overall, our study suggests enhanced adaptation of Cry1Ab-resistant larvae on transgenic Cry1Ab corn as revealed by lower number and lower ratios of differentially expressed genes in R-strain than in S-strain of *O. nubilalis*.

## 1. Introduction

Transgenic crops expressing insecticidal proteins from the bacterium *Bacillus thuringiensis* (Bt) have been adopted in insect pest management programs worldwide [1]. For example, transgenic Bt corn has successfully managed devastating insect pest, the European corn borer (*Ostrinia nubilalis* Hübner) [2]. However, insects have a remarkable ability to adapt and develop resistance to Bt toxins and this is considered the main threat to the continued success of Bt crops in the field [3]. In fact, field evolved resistance to Bt crops has already been reported in *Spodoptera frugiperda*, *Pectinophora gossypiella*, *Diabrotica virgifera virgifera* [4] and *Helicoverpa armigera* [5]. Although field evolved resistance to Bt corn has not been reported in *O. nubilalis*, selection with Dipel-ES (including several Bt protoxins) resulted in 65-fold resistance to Cry1Ab (LC_50_) after seven generations [6], and selection with Cry1F protoxin resulted in >3000-fold resistance [7].

The most common Bt resistance mechanisms include the changes in proteolytic processing of Cry toxins [8] and changes in binding of Bt toxins to midgut receptors [9]. For example, a Dipel-selected strain of *O. nubilalis* was found to be associated with reduced protease activity and its resistance can be virtually abolished when resistant insects were fed on transgenic Bt corn (MON810-event) [6,10]. Similar resistance mechanisms have been found in other insect species [11]. Changes of Bt receptors, such as cadherin-like protein (Cad), aminopeptidase N (APN) and alkaline phosphatase (ALP) may also lead to Bt resistance in insects. A highly variable Cad protein (caused by alternative splicing) was found to associate with field-evolved resistance to Bt cotton in *P. gossypiella* [12], and a deletion mutation in the aminopeptidase N1 gene was related to Cry1Ac resistance in *H. arimgera* [13]. In addition, reduced abundance or binding of receptors for Cry1F has been found to associate with Cry1F resistance in *S. frugiperda* [14,15,16]. Furthermore, cellular signaling pathways and pore-formation may interact with each other in Bt resistance development. For example, the mitogen-activated protein kinase pathway (MARK signaling pathway) appeared to alter the expression of ALP and ATP-binding cassette transporter subfamily C (ABCC) genes in the gut of a Cry1Ac resistant strain of *Plutella xylostella* [17].

Theoretically, any changes in gut proteins that are associated with Bt toxicity, including proteases, membrane binding partners and intracellular defense molecules either through differential expression, alternative splicing, silencing or mutation, could potentially confer Bt resistance. However, differences in gut transcriptional responses to the ingestion of transgenic plants expressing a Bt toxin have not been systematically examined in Bt-resistant and susceptible strains of an insect species. In this study, we used microarrays to compare gut gene transcriptional responses between Cry1Ab-resistant and susceptible strains of *O. nubilalis* when larvae were fed transgenic Cry1Ab corn leaves. We hypothesized that Cry1Ab-resistant and susceptible strains of *O. nubilalis* larvae might respond differently in transcription of gut genes when larvae were fed on the leaves of transgenic corn expressing Cry1Ab toxin, and such differential expression profiles may also potentially help us better understand how insects may adapt transgenic Bt crops by regulating gut gene expression and reveal candidate genes contributing to Bt toxicity and/or resistance in insects.

## 2. Results and Discussion

### 2.1. Comparison of Susceptibilities of S- and R-Strain Larvae to Cry 1Ab Protoxin and Transgenic Cry1Ab Corn Leaves

The LC_50_ of R-strain was 48 μg/mL diet whereas the LC_50_ of S-strain was 0.2 μg/mL diet (Table 1). Resistance ratio of R-strain reached 220 folds to Cry1Ab protoxin. Even though third-instar larvae were resistant by more than 200 folds to Cry1Ab protoxin, they were not able to survive on transgenic corn leaves. The LT_50_ for the early third-instar larvae of R- and S-strains were 5.4 and 3.6 days, respectively (Table 1) and no mortality was found in either R- or S-strain larvae fed non-transgenic corn leaves expressing Cry1Ab toxin. The previous study in our laboratory also showed that both S and R-strain larvae maintained similar susceptibility to Bt corn [10].

### 2.2. Gene Expression Profiles of S- and R-Strain Larvae Fed Transgenic Corn Leaves

**Figure 1:**
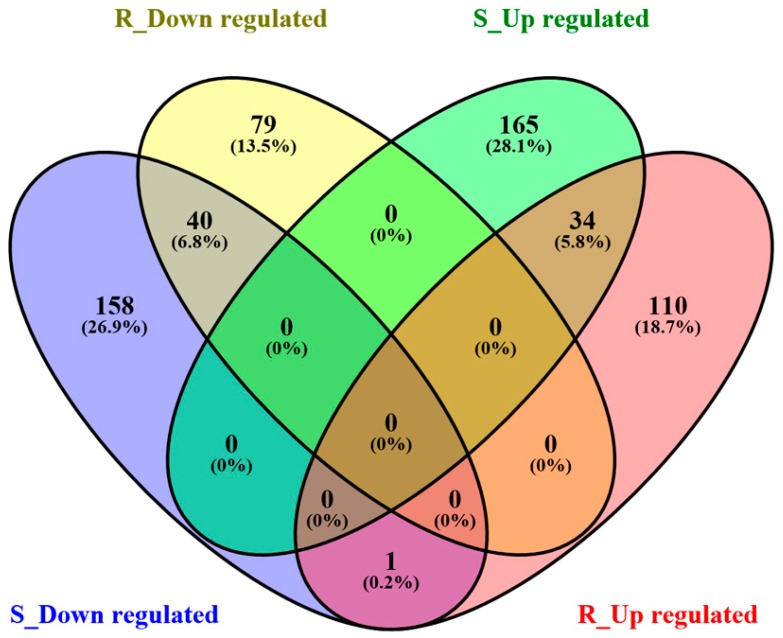
A Venn diagram showing the numbers of up- and down-regulated gut genes in S- and R-strain larvae of *O. nubilalis* fed transgenic corn leaves expressing Cry1Ab toxin as compared with those fed non-transgenic corn leaves. S_Down regulated (slate blue), S_Up regulated (green), R_Down regulated (yellow), and R_Up regulated (salmon) denote down- and up- regulated gut genes in S- and R-strains of *O. nubilalis* larvae fed transgenic corn leaves expressing Cry1Ab for 6 h, respectively.

When the larvae fed Cry1Ab transgenic corn leaves were compared with those fed non-transgenic corn leaves, 398 out of 2895 gut genes showed significantly differential expression in S-strain, but only 264 gut genes showed significantly differential expression in R strain (expression ratio ≥2.0 with *p* value <0.05) (Figure 1). This reflects approximately 34% more gut genes with significant expression changes in S-strain than in R strain. This implies that the larvae of R-strain have better adapted to Cry1Ab toxin than the larvae of S-strain because fewer gut genes in R-strain changed the expression when the larvae ingested transgenic corn leaves expressing Cry1Ab toxin.

Of the 398 gut genes in S-strain that were differentially expressed when exposed to transgenic corn leaves expressing Cry1Ab toxin, 199 were up-regulated and 199 were down-regulated. Of the 264 gut genes in R-strain that were differentially expressed when exposed to transgenic corn leaves, 145 were up-regulated and 119 were down-regulated. In these two strains, 40 down-regulated gut genes and 34 up-regulated gut genes were in common between the two strains (Figure 1). Although the percentages of down-regulated genes among the total number of differentially expressed genes (199 genes or 50% in S-strain; 119 genes or 45% in R-strain) were similar between R- and S-strains, the expression ratios of down-regulated genes were much higher in S-strain than in R-strain (Table 2). For example, contig[0243], a trypsin-like gene (Accession No: AFM77760), was significantly down-regulated in both strains and the expression ratio in S-strain was almost twice as large as in R-strain. The possible biological functions of differentially expressed genes were further examined by searching for descriptions of their homologs in the gene databases (Table S1). However, nearly one third of these transcripts (122 out of 398 in S-strain and 95 out of 264 in R-strain) did not have detailed functional annotations.

Yao et al. [18] reported that when S-strain larvae were fed an artificial diet containing Cry1Ab protoxin, 174 gut genes showed significantly differential expressions. Among these genes, 94 were up-regulated and 82 were down-regulated. If we compare the numbers of differentially expressed gut genes between S-strain larvae fed artificial diet containing Cry1Ab protoxin and S-strain larvae fed transgenic corn leaves expressing Cry1Ab toxin, 75 gut genes (27 + 48) were in common (Figure S1). However, if we compare the numbers of differentially expressed genes among S-strain larvae fed artificial diet containing Cry1Ab protoxin, and S- and R-strain larvae fed transgenic corn leaves expressing Cry1Ab toxin, 48 gut genes were in common (Figure S1). This suggests that these 48 genes may be directly or indirectly involved in cell protection during Cry1Ab attack (Table S2). For example, several genes, such as contig[0814], contig[0009], and contig[4527], were down-regulated in all the three treatments, and the highest down-regulation ratios were found in S-strain larvae fed transgenic corn leaves, whereas the lowest down-regulation ratios existed in R-strain larvae fed transgenic corn leaves. These results also suggested that the change of gut gene expression in R-strain larvae was less responsive to the ingestion of transgenic corn expressing Cry1Ab toxin than that of S-strain larvae, which could be explained by the adaptation of R strain larvae to Cry1Ab toxin.

### 2.3. Gut Genes Potentially Involved in Cry1Ab Toxin Activation or Degradation

**Figure 2:**
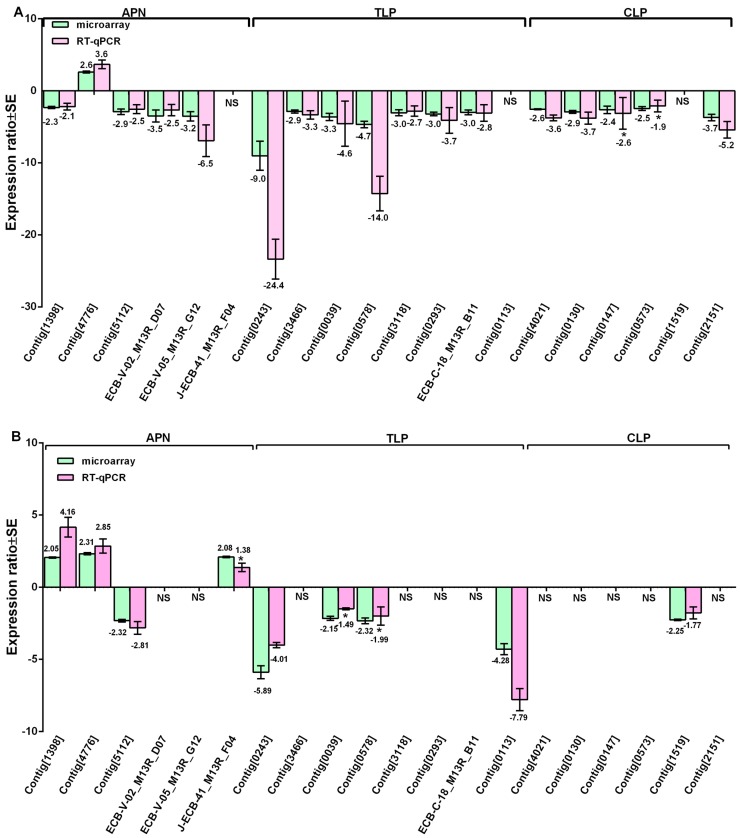
Comparison of microarray (green bars) and reverse transcription quantitative PCR (RT-qPCR) (pink bars) analyses for three functional groups of the gut genes with significantly differential expressions in S-strain (**A**) and R-strain (**B**) of *O. nubilalis* larvae fed transgenic Cry1Ab corn leaves relative to non-transgenic corn leaves. Aminopeptidase (APN), trypsin-like proteases (TLP), and chymotrypsin and chymotrypsin-like proteases (CLP). “NS” indicates the gene did not show significantly differential expression. The symbol “*” indicates that RT-qPCR of the genes did not show significant difference between transgenic corn Cry1Ab and non-transgenic corn treatments (*p* > 0.05).

Serine protease-mediated Bt resistance has been documented in many studies [19,20,21]. In our current study, the gut genes encoding serine proteases-like trypsins and chymotrypsins, were all down-regulated in both R- and S-strain larvae fed transgenic corn leaves expressing Cry1Ab toxin (Figure 2). These down-regulated serine protease-like genes in S-strain larvae included contig[0130], contig[0147], contig[4021], contig[0573], contig[0243], contig[0039], contig[3466], contig[0578], contig[3118], contig[0293], contig[2151], and ECB-C18-B11 (Figure 2A). Two serine protease-like genes (contig[0243], ECB-C-18_B11) were also down-regulated in S-strain larvae fed Cry1Ab protoxin (Table S2). However, the level of down-regulation for contig[0243] was approximately 2.7-fold higher in the larvae fed transgenic Cry1Ab corn leaves than those fed Cry1Ab protoxin.

In contrast, four serine protease-like genes (contig[0389], contig[1207], contig[3704], and contig[4768]) were up-regulated more than 5-fold in S-strain larvae fed Cry1Ab protoxin [18], but were not differentially expressed in S-strain larvae fed transgenic corn leaves. In addition, three serine protease-like genes (contig[0243], contig[0039], contig[0578]) were down-regulated in both strains (Figure 2A,B), but their changes of expression ratios in R-strain larvae were much lower than in S-strain larvae. Overall, the change in expression ratios of serine protease genes appeared to be more dramatic in S-strain larvae than in R-strain larvae fed on transgenic Cry1Ab corn leaves. These results provide genetic evidences of larval adaptation to Cry1Ab toxin in R strain.

### 2.4. Gut Genes Might Be Potentially Involved in Toxin Binding

**Figure 3:**
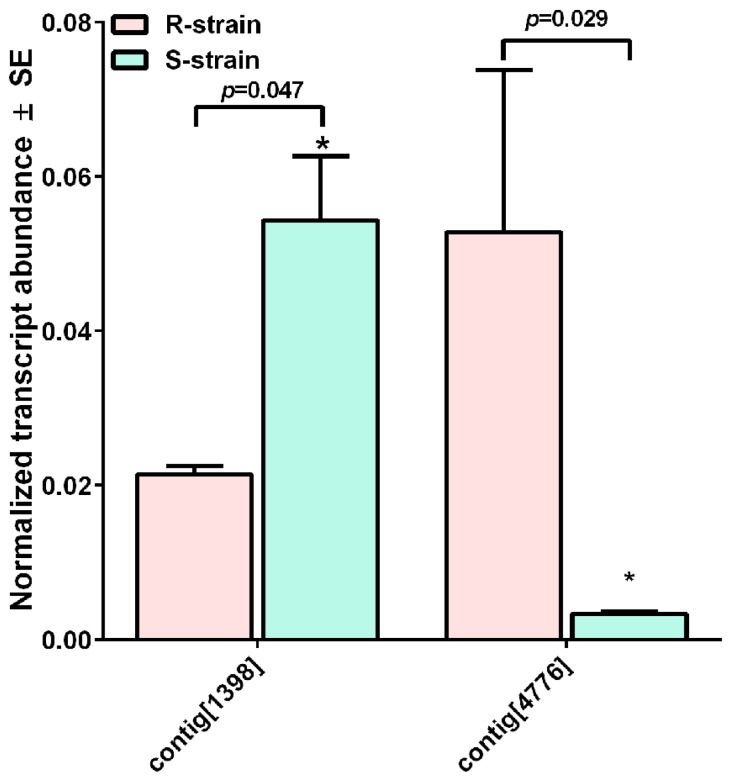
The transcriptional abundances of two aminopeptidase genes (contig[1398] and contig[4776]) in larval guts of R-strain (red) and S-strain (green) in the absence of Cry1Ab exposure as evaluated by RT-qPCR. The symbol “*”denotes statistical difference at 0.05 between R- and S-strain larvae (Student’s *t*-test).

We did not find any significant difference in the expression of cadherin gene in S- and R-strain larvae. However, we found that four APN genes (contig[1398], contig[5112], ECB-V02-D07 and ECB-V05-D12) were significantly down-regulated and one APN gene (contig[4776]) was up-regulated in S-strain larvae fed transgenic corn leaves (Figure 2A). In contrast, we found that three APN genes (contig[1398], contig[4776], and J-ECB-41_F04) were significantly up-regulated and one APN gene (contig[5112]) was down-regulated in R-strain larvae fed transgenic corn leaves (Figure 2B). We suggest that the up-regulated APN genes (e.g., contig[4776]) might be involved in degradation of Cry1Ab toxin whereas down-regulated genes (e.g., contig[5112]) might play an important role in the Cry1Ab binding as reduced expression of Cry1Ab binding receptors can reduce the toxicity of Cry1Ab to insects. It should also be pointed out that the expression level of contig[1398] was 2.5-fold lower but that of contig[4776] was 17-fold higher in R-strain larvae than in S-strain larvae even if these larvae were not exposed to Cry1Ab (Figure 3).

Xu et al. [22] identified APN-N1 in *O. furnacalis* (GenBank accession No: ACX85727), which shares 98% identity with contig[1398], as a Cry1 toxin resistance gene with lower expression in *O. furnacalis* and *H. armigera*. They also reported that APN-N2 of *O. furnacalis* (GenBank accession No: ACB47287), which shares 95% identity with contig[4776], was involved in Bt toxicity in *O. furnacalis*. Other studies also showed that silencing of aminopeptidase genes by RNAi resulted in decreased susceptibility to Cry toxin in *O. nubilalis* and *S. exigua* [23]. Thus, these studies also support our notion that contig[4776] and contig[1398] could play roles in *O. nubilalis* resistant to Cry1Ab. Further research would be necessary to reveal biological roles of these APN genes in the degradation of Cry1Ab toxin and in the reduction of Cry1Ab binding in *O. nubilalis*.

### 2.5. Transcriptional Responses of Other Genes Potentially Involved in Larval Defense to Bt Toxins

Five out of six genes in S-strain and three out of the six genes in R-strain, potentially involved in the biosynthesis and metabolism of chitin and the formation of gut peritrophic matrix, were significantly down-regulated in larvae fed transgenic Cry1Ab corn leaves. These genes include chitin deacetylase 2 (contig[0233]), chitin deacetylase 5b (contig[0505]), chtinase 8 (contig[0188]), peritrophic matrix chitin binding protein (contig[4654]), glucosamine-fructose-6-phosphate aminotransferase 2 (ECB-C-05_D05), and fructose-6-phosphate aminotransferase 2 (contig[4598]) (Table S1). Although many of these genes are involved in chitin biosynthesis and metabolism, which can’t be easily related to larval defense to Cry1Ab, it is well-known that chitinases are major enzymes that degrade chitin. The down-regulation of chitinase genes is likely to avoid weakening the gut peritrophic matrix of insects [24,25]. Thus, the down-regulation of chitinase gene may serve as a defense mechanism against Cry toxins by maintaining the integrity of the gut system.

Our study also revealed a significant up-regulation of a heat shock protein (HSP) 70 gene (contig[0227]) in both S- and R-strain larvae fed transgenic Cry1Ab corn leaves. Hsp70, Hsp90 and Hsp60, which have been identified as markers of stress-related responses in insects, can be induced by many different stressors. These genes may also function in gene-regulation networks to maintain cell homeostasis [26], and have been considered as defense factors against bacterial and virus infection in arthropods [27,28]. Indeed, Hsp70 has been well-characterized as a stress-induced gene in the silk worm [29]. In the castor semi-looper, (*Achaea janata*), the exposure to Cry toxin, as considered as an oxidative stress, also induced the expression of several genes encoding antioxidant enzymes [30]. Based on these studies, it is likely that the up-regulation of Hsp70 in both S- and R-strain larvae fed transgenic Cry1Ab corn leaves may be related to the stress posed by Cry1Ab toxin and could be used as an indicator of Cry1Ab exposure in *O. nubilalis*.

### 2.6. Summary and Possible Constraints of the Study

This study identified differentially expressed gut genes when *O. nubilalis* larvae were fed transgenic corn leaves expressing Cry1Ab toxin. Specifically, more gut genes (398) changed their expression levels in S-strain (398) than in R-strain (264). The ratios of differentially expressed genes were also much higher in S-strain than in R-strain (Table 2). Many of these differentially expressed genes appear to be related to Bt toxicity and resistance [31]. Overall, these results provide genetic evidences of larval adaptation to Cry1Ab toxin when R-strain larvae ingested transgenic corn leaves expressing Cry1Ab toxin. Our research represents the first systematic study by using genomic approaches to compare transcriptional responses of the gut genes in a major agricultural insect pest fed transgenic corn leaves expressing Cry1Ab toxin and its non-transgenic corn isoline. Such expression profiles may help us better understand how insects may adapt transgenic Bt crops by regulating gut gene expression and reveal candidate genes contributing to Bt toxicity and/or resistance in insects.

However, care must be taken when we explain insect responses based on the differential gene expression data from the larvae fed transgenic corn leaves expressing Cry1Ab toxin and non-transgenic corn leaves. We chose a 2-fold difference in expression as indicative of a response to Cry1Ab; however, this cut-off is arbitrary. It is possible that the differentially expressed genes between R- and S-strain larvae fed each of the two corn lines could be attributed to possible differences unrelated to either Bt toxicity and resistance. This contention was supported by the large number of differentially expressed genes between S- and R-strain larvae fed non-transgenic corn leaves (Table S3). For example, among 15 serine protease and serine protease-like gut genes, 14 showed decreased expression in R-strain as compared with S-strain. Most of these genes showed low levels of differential expression. However, three of these genes, ECB-V-27_E08, J-ECB-50_G05 and contig[1207], displayed 23.76-, 42.35- and 14.06-fold, respectively, decreased expression in R-strain larvae. These genes could be potentially involved in resistance to Cry1Ab protoxin through a reduced activation of the protoxin to Cry1Ab toxin as R-strain was originally selected with Cry1Ab protoxin [32]. In contrast, a multidrug resistance 1A-like gene (J-ECB-33_E10) showed 59.13-fold higher expression in R-strain than in S-strain. Such a dramatically increased expression of this gene in R-strain as compared with that of S-strain may suggest its involvement in Bt resistance. Multidrug resistance proteins are ATP-binding cassette subfamily B proteins which can contribute to insect resistance to multiple Bt toxins [33].

It is also possible that some of differentially expressed genes in R-strain or S-strain larvae fed the two corn lines could be attributed to possible differences in plant physiology (e.g., expression levels of plant nutrients, antinutrients, and other metabolites) between the two corn lines although they are near isolines. If such physiological differences between the two corn lines exist, they may also modify the levels of differential gene expression in R- or S-strain larvae fed the two corn lines. Additionally, selection for Cry1Ab-resistance over 137 generations may have led to accumulation of multiple genetic changes, especially as the level of exposure to Bt was increased over time. Low levels of Bt exposure in early generations may have selected for certain genetic differences that enable the insects to overcome weak intoxication (e.g., detoxification mechanisms, reduced expression of receptors), whereas high exposure later in selection may have caused stronger mechanisms of resistance. Thus, it is not possible from the present research to determine what gene expression differences would be relevant under field conditions, where *O. nubilalis* larvae are exposed to a high dose of Cry1Ab in plants.

It is also difficult to distinguish gene expression differences that are related to a mechanism of resistance from those that are related to the effects of intoxication. As resistant insects are less affected by Cry1Ab toxicity, defense mechanisms would be expected to not be activated, or activated at lower levels. For example, most genes in Table 2 responded very similarly in R- and S-strains. These genes may represent a response to exposure to Bt but not to resistance or to toxicity. The subset that respond less in R- than in S-strain but in same direction (e.g., carboxylesterases/carboxypeptidases, some transporters, sterol desaturases, alcohol dehydrogenase, carbonyl reductase, cytochrome b561-like, peroxisomal membrane protein, cystathionine γ-lysase), could be related to a physiological response to intoxication in S-strain that are not triggered in R-strain.

In addition, further studies will be necessary to validate or clarify functional roles of differentially expressed genes in Cry1Ab toxicity and resistance. Clearly, RNAi would be a logical approach for follow-up studies. Currently, however, lepidopteran insects, such as *O. nubilalis*, have not been a taxonomic group with robust RNAi responses, which limits our follow-up studies to validate or clarify functional roles of differentially expressed genes in *O. nubilalis*.

## 3. Materials and Methods

### 3.1. Insect Larvae Rearing and Transgenic Corn Planting

A Bt-resistant strain (R-strain, Sky) and its susceptible isoline strain (S-strain, Meads) of *O. nubilalis* were kindly provided by Blair Siegfried at the University of Lincoln-Nebraska [32], in which R-strain had been selected from S-strain by feeding them an artificial diet containing Cry1Ab protoxin (0.3 µg/mL) for 137 generations in the laboratory. The eggs were collected from wax paper each day and kept in insect rearing cups which provided high humidity (above 80%) until they hatched. Newly hatched larvae were transferred to artificial diet and reared to the third instar for testing. The larval developmental stage was determined by transferring individuals to a new rearing dish after each molt.

The transgenic Cry1Ab corn (Pioneer 34P88 with MON810) and its isoline (Pioneer 34P86) (Pioneer Hybrid Inc., Johnston, IA, USA) were planted in the greenhouse and grown until the six-leaf stage at Kansas State University, Manhattan, KS, USA. Fresh corn leaves were collected daily and cut into ca. 4-cm^2^ pieces and were fed to the larvae.

### 3.2. Bioassays of Cry 1Ab Protoxin and Transgenic Cry1Ab Corn Leaves

To assess the resistance level of R-strain larvae to Cry1Ab protoxin for gene expression analysis, LC_50_ values of Cry1Ab protoxin were determined in both R- and S-strains according to previously described studies [18]. Each bioassay was conducted with more than 360 third-instar larvae that were fed artificial diet containing Cry1Ab protoxin at 0, 0.3, 3, 10, 25, 50, 100, and 234 μg/mL. Each concentration was replicated three times with an average of 16 larvae in each replicate. To ensure that the larvae actually fed the treated diet, they were starved for 24 h and then transferred into rearing cups with the diet containing Cry1Ab protoxin. Mortality was recorded after seven days. The LC_50_ values were analyzed for R- and S-strains by probit analysis using Statistical Analysis System (SAS Institute, Cary, NC, USA). Previous studies showed that the LC_50_ values of Cry1Ab activated toxin and Cry1Ab protoxin for R-strain were essentially the same [34]. Therefore, we didn’t repeat the bioassays in the current study.

To evaluate the resistance level of R-strain to transgenic corn expressing Cry1Ab toxin, bioassays were conducted by feeding early third-instar larvae of both R- and S-strains with transgenic corn leaves at 26 °C and 16L:8D photoperiod. In this experiment, all tested larvae were starved for 24 h before they were individually transferred into rearing cups with transgenic or non-transgenic corn leaves (one larva per rearing cup). The bioassay for each strain was replicated three times and each replicate included approximately 50 larvae. The fresh leaves were replaced daily and the survival was recorded daily for one week. The LT_50_ values for both R- and S-strains were calculated by probit analysis using Statistical Analysis System (SAS Institute).

### 3.3. Microarray Analysis

Forty early third-instar larvae of each strain were starved for 24 h and then individually transferred to 20 rearing cups containing transgenic corn leaves and 20 rearing cups containing non-transgenic isoline corn leaves. After feeding for 6 h, guts of these larvae were dissected and five guts were pooled as one sample. Total four replicates (four samples) of each treatment were obtained for total RNA extraction. The experiment included two treatments (S- and R- strains), two corn materials (Cry1Ab transgenic and non-transgenic corn leaves) and four replicates for a total of 16 biological samples. Total RNA for each same was extracted using TRIzol reagents (Invitrogen, Frederick, MD, USA). RNA quantity and quality were determined using a NanoDrop 2000 Spectrophotometer (Thermo Fisher Scientific, Wilmington, DE, USA) and an Agilent 2100 bioanalyzer, respectively.

Five oligonucleotide probes from each of 2895 unique genes from *O. nubilalis* larval gut [35] were computationally designed using Agilent’s probe design algorithms (Agilent, Santa Clara, CA, USA). Two Agilent custom microarray slides (each containing 8 microarrays) for a total of 16 microarrays were used to compare gut transcriptional responses in the larvae of R- and S-strains after fed transgenic and non-transgenic corn leaves. Each microarray was tested with 12,972 probes representing 2895 unique larval gut genes of *O. nubilalis*. The microarrays on each slide were randomly assigned a feeding treatment and replication. The signal intensity of each hybridized spot on array slides was qualified and quantified with Agilent Feature Extraction Ver. 9.5 software (Agilent). The raw data files with 12,972 customer designed probes and control probes extracted by Agilent Feature Extraction software were imported to GeneSpring GX11 for normalization algorithm [18]. The expression of each gene for each strain was expressed as the ratio between the transgenic corn leave-fed group and the non-Bt leave-fed group for resistant and susceptible strains, respectively. These ratios were then tested statistically at a *p* value <0.05 (one-way ANOVA) with expression ratio ≥2.0 as significant. Gene ontologies for those significantly differential expressed genes (i.e., either up or down-regulated genes with expression ratio ≥2.0 at a *p* value <0.05) were further analyzed by using Blast2go (available on: http://www.blast2go.org) at level 2. The datasets of the gene expression profiles have been deposited in the NCBI Gene Expression Omnibus (GEO) repository with the accession number of GSE87393 (available on: http://www.ncbi.nlm.nih.gov/geo/query/acc.cgi?acc=GSE87393).

### 3.4. Validation of Expression Changes by RT-qPCR

All candidate gut genes with significantly differential expressions were identified based on the model of action of Cry toxin-induced pore formation. The expression ratio of each candidate gene was also validated further by reverse transcription quantitative PCR (RT-qPCR). In this validation, 1.0 µg of total RNA from each microarray replicate sample was reverse-transcribed in a 20-μL reaction mixture with Fermatas ReverAid™ First Strand cDNA synthesis kit (Fermentas, Glen Burnie, MD, USA). The specific primers for 20 selected genes and the reference gene ribosome protein L18 (*RPL18*) were designed using Beacon 7 Designer™ (Primer Biosoft, Palo Alto, CA, USA) (Table 3). RT-qPCR was performed with 2-step amplification protocol (40 cycles of 95 °C for 30 s, 56 °C for 30 s) on a Bio-Rad iCycler (Bio-Rad Laboratories, Hercules, CA, USA) using Fermentas SYBR green qPCR kit. To make comparisons of expression levels of the selected gut genes, the abundance of each gene was normalized to *RPL18* after feeding on transgenic and non-transgenic corn [18]. The expression ratio of each gene was calculated using the normalized gene expression abundance for insect guts from larvae fed transgenic corn leaves divided by the expression abundance for insect guts from larvae fed non-transgenic corn leaves.

## 4. Conclusions

We identified differentially expressed gut genes after Cry1Ab-susceptible and resistant larvae of *O. nubilalis* were fed transgenic corn leaves expressing Cry1Ab toxin. More gut genes changed their expression levels in S-strain than in R-strain. The ratios of differentially expressed genes were also much higher in S-strain than in R-strain. Many of these differentially expressed genes appear to be related to Bt toxicity and resistance. Our study strongly suggests enhanced adaptation of Cry1Ab-resistant larvae on transgenic Cry1Ab corn as revealed by lower number and lower ratios of differentially expressed genes in R-strain than in S-strain. However, care must be taken when we explain insect responses based on the differential gene expression data because of other unknown variables as discussed in this paper. Further studies will be necessary to validate or clarify functional roles of differentially expressed genes involved in Cry1Ab toxicity and resistance in *O. nubilalis*.

## Figures and Tables

**Table 1:** LC_50_ (μg/mL) and LT_50_ (days) data from probit analysis for R- and S-strains of *O. nubilalis* larvae fed Cry1Ab protoxin and transgenic corn leaves expressing Cry1Ab toxin (Cry1Ab corn), respectively.

Treatment	Strains	Number of Larvae	Slope and SE	LC_50_/LT_50_ (95% CI)	*Chi*-Square	*p*-Value > *Chi-*Sq	Resistance Ratio
Cry1Ab protoxin	R	365	0.61 ± 0.12	48.47 μg/mL (30.18–78.43)	10.62	0.94	220
	S	365	1.24 ± 0.15	0.22 μg/mL (0.14–0.33)	29.56	0.058	
Cry1Ab corn	R	144	0.74 ± 0.06	5.38 days (4.79–6.00)	42.06	0.16	-
	S	175	1.32 ± 0.16	3.68 days (3.47–3.88)	22.39	0.13	-

**Table 2:** List of gut genes in S- and R-strain larvae of *O. nubilalis* with a significantly differential expression after fed transgenic Cry1Ab as compared with those fed non-transgenic (control) corn leaves for 6 h.

EST ID	GenBank EST ID ^#^	Sequence Description	*E*-Value	GenBank Homolog	Expression Ratios ± SE *	
					S:Plant	R:Plant
**Trypsin, chymotrypsin, serine protease inhibitors and cysteine B like proteases**						
Contig[0039]	GH997464.1	trypsin serine protease (*Ostrinia furnacalis*)	0.0	AAX62032	−3.63 ± 0.28	−2.01 ± 0.01
Contig[0243]	GH998064.1	trypsin-like serine protease 12 (*Ostrinia nubilalis*)	1 × 10^−174^	AFM77760	−9.02 ± 0.89	−5.89 ± 0.45
Contig[0578]	GH996481.1	chymotrypsin-like protease (*Helicoverpa armigera*)	6 × 10^−124^	AFW03964	−4.68 ± 0.32	−2.32 ± 0.20
**Aminopeptidase**						
Contig[1398]	GH993761.1_1605111	aminopeptidase N isoform 1 (*Ostrinia furnacalis*)	0.0	ACJ64827	−2.32 ± 0.12	2.05 ± 0.04
Contig[4776]	GH998970.1	Cry1Ab-RR resistance protein APN2 (*Ostrinia furnacalis*)	0.0	ACJ64828	2.59 ± 0.08	2.31 ± 0.07
Contig[5112]	GH997440.1	aminopeptidase N 3a (*Ostrinia nubilalis*)	0.0	ADA57169	−2.90 ± 0.17	−2.32 ± 0.10
**Carboxylesterase and carboxypeptidase**						
Contig[5691]	GH990344.1	carboxyl/choline esterase (*Helicoverpa* ar*m*igera)	6 × 10^−56^	ADJ96632	−10.91 ± 0.52	−4.57 ± 0.09
ECB-V-29_E10	GH996536.1	midgut carboxypeptidase 2 (*Danaus plexippus*)	3 × 10^−20^	EHJ75106	−17.64 ± 2.56	−4.19 ± 0.35
Contig[0009]	GH992549.1	zinc carboxypeptidase A 1 (*Danaus plexippus*)	8 × 10^−78^	EHJ72811	−3.85 ± 0.22	−2.09 ± 0.01
Contig[0019]	GH998697.1	plasma glutamate carboxypeptidase (*Danaus plexippus*)	3 × 10^−106^	EHJ64655	−6.65 ± 0.31	−2.28 ± 0.04
**Chitin related transcripts**						
Contig[0505]	GH991666.1	chitin binding PM protein (*Helicoverpa armigera*)	0.0	ABU98616	−3.30 ± 0.35	−2.43 ± 0.04
Contig[0233]	GH989367.1	chitin deacetylase 2 (*Mamestra brassicae*)	1.25 × 10^−66^	AEI30869	−2.60 ± 0.10	−2.44 ± 0.06
ECB-C-05_D05	GH992955.1	glucosamine-fructose-6-phosphate aminotransferase 2 (*Culex cuinquefasciatus*)	6.25 × 10^−21^	XP_001848160	−2.88 ± 0.07	−2.10 ± 0.03
**Transcription regulator factors**						
ECB-09_F07	GH997837.1	Rho GTPase-activating protein 12-like (*Acyrthosiphon pisum*)	1.68 × 10^−65^	EHJ79227	2.70 ± 0.18	2.76 ± 0.19
ECB-V-14_D07	GH995295.1	Reverse transcriptase (*Ostrinia nubilalis*)	5 × 10^−11^	ABO45233	2.30 ± 0.09	2.22 ± 0.04
**Heat shock protein**						
Contig[0227]	GH997898.1	heat shock cognate 70 (*Plutella xylostella*)	0.0	BAE48743	2.36 ± 0.14	2.06 ± 0.03
**Transporter**						
Contig[0814]	GH998546.1	sodium-bile acid cotransporter (*Danaus plexippus*)	1 × 10^−27^	EHJ73754	−14.14 ± 0.62	−4.21 ± 0.26
Contig[1314]	GH993616.1	putative amino acid transporter (*Danaus plexippus*)	2 × 10^−73^	EHJ64672	−3.80 ± 0.14	−2.52 ± 0.16
Contig[4763]	GH998142.1	sodium-bile acid cotransporter (*Aedes aegypti*)	2 × 10^−65^	XP_001662576	−10.83 ± 2.43	−6.56 ± 0.40
Contig[5496]	GH998547.1	putative sugar transporter (*Danaus plexippus*)	2 × 10^−103^	EHJ70957	−2.66 ± 0.19	−2.36 ± 0.10
Contig[5743]	GH993678.1	putative amino acid transporter (*Danaus plexippus*)	7 × 10^−97^	EHJ64672	−3.96 ± 0.22	−2.55 ± 0.07
ECB-16_F07	GH998441.1	sodium-dependent phosphate transporter (*Danaus plexippus*)	4.45 × 10^−57^	EHJ78425	−5.51 ± 0.60	2.83 ± 0.13
ECB-21_C09	GH998857.1	sugar transporter (*Culex quinquefasciatus*)	6.29 × 10^−30^	EHJ73890	−4.90 ± 0.24	−2.12 ± 0.06
ECB-V-22_E06	GH995942.1	putative GDP-fucose transporter (*Danaus plexippus*)	7 × 10^−134^	EHJ67364	2.02 ± 0.01	2.39 ± 0.02
J-ECB-39_E12	GH992066.1	sugar transporter (*Anopheles darlingi*)	3.41 × 10^−15^	ETN58527	−9.56 ± 1.92	−4.35 ± 1.13
J-ECB-52_H10	GH988032.1	putative monocarboxylate transporter (*Danaus plexippus*)	3.05 × 10^−17^	EHJ67333	−3.30 ± 0.27	−2.13 ± 0.07
J-ECB-55_E04	GH988996.1	monocarboxylate transporter 9-like (*Bombyx mori*)	6.00 × 10^−38^	XP_004927805	−3.62 ± 0.24	−3.88 ± 0.10
**Xenobiotics detoxification enzyme**						
Contig[0004]	GH992504.1	glutathione S-transferase (*Choristoneura* *fumiferana*)	4.79 × 10^−52^	AAF23078	−8.76 ± 1.41	−2.95 ± 0.11
**Other metabolic enzymes**						
Contig[0923]	GH999509.1	neutral lipase (*Helicoverpa armigera*)	7.84 × 10^−80^	AFI64310	−7.96 ± 0.50	−10.45 ± 0.46
Contig[1081]	GH998825.1	neutral lipase (*Helicoverpa armigera*)	6.98 × 10^−55^	AFI64314	−4.83 ± 0.22	−5.02 ± 0.02
Contig[1486]	GH997709.1	c-5 sterol desaturase erg32-like (*Bombyx mori*)	1.25 × 10^−63^	XP_004922936	−7.75 ± 0.48	−2.80 ± 0.28
Contig[1897]	GH997709.1	c-5 sterol desaturase erg32-like (*Bombyx mori*)	9.20 × 10^−92^	XP_004922936	−7.03 ± 0.05	−2.13 ± 0.02
J-ECB-61_C03	GH987254.1	UDP-glycosyltransferase UGT40K1 (*Bombyx mori*)	7.52 × 10^−47^	AEW43171	2.57 ± 0.14	2.08 ± 0.02
J-ECB-15_G11	GH990690.1	putative 6-phosphofructo-2-kinase/fructose-2,6-biphosphatase 4 (*Danaus plexippus*)	3.69 × 10^−43^	EHJ64247	2.65 ± 0.17	2.42 ± 0.06
Contig[4515]	GH987646.1	putative γ-glutamyl hydrolase (*Danaus plexippus*)	2.23 × 10^−70^	EHJ77729	−2.56 ± 0.17	−2.49 ± 0.03
BM2_B12	GH992538.1	estradiol 17-β-dehydrogenase 8-like isoform X1 (*Bombyx mori*)	2.23 × 10^−48^	XP_004928638	−2.24 ± 0.03	−2.30 ± 0.24
Contig[0022]	GH992601.1	alcohol dehydrogenase (*Danaus plexippus*)	2.89 × 10^−53^	EHJ65258	−6.41 ± 0.79	−2.36 ± 0.05
Contig[1778]	GH994839.1	glutaryl-CoA dehydrogenase (*Bombyx mori*)	1.56 × 10^−57^	XP_004932115	−2.50 ± 0.05	−2.63 ± 0.05
gi_133906638	EL929475.1	retinol dehydrogenase 11-like (*Bombyx mori*)	8.18 × 10^−30^	XP_004926801	−10.53 ± 0.93	−10.95 ± 1.40
Contig[0362]	GH997850.1	carbonyl reductase [NADPH] 3-like (*Bombyx mori*)	2.85 × 10^−89^	XP_004929128	−4.82 ± 0.30	−2.14 ± 0.05
**Others**						
Contig[0218]	GH997574.1	NADPH cytochrome b5 reductase (*Spodoptera exigua*)	1.48 × 10^−117^	ADX95747	−2.14 ± 0.01	−2.02 ± 0.01
Contig[5679]	GH988679.1	phosphoserine aminotransferase (*Antheraea pernyi*)	3.47 × 10^−81^	ADO79970	−4.08 ± 0.08	−3.26 ± 0.14
Contig[0535]	GH990867.1	hypothetical protein RR46_06729 (*Papilio xuthus*)	5 × 10^−38^	KPI94278	−2.71 ± 0.11	−2.53 ± 0.43
Contig[1573]	GH998860.1	lipopolysaccharide-induced tumor necrosis factor (*Bombyx mori*)	9.41 × 10^−14^	XP_004928093	2.28 ± 0.07	2.76 ± 0.22
Contig[1868]	GH995418.1	ER protein reticulon (*Aedes aegypti*)	1.16 × 10^−43^	ABF18334	2.74 ± 0.15	2.21 ± 0.02
Contig[1880]	GH995131.1	adipokinetic 3 (*Helicoverpa armigera*)	1.46 × 10^−06^	AGH25546	5.46 ± 1.17	4.09 ± 0.70
Contig[1953]	GH997798.1	prostaglandin reductase 1-like (*Papilio xuthus*)	3 × 10^−145^	XP_013169440	−2.71 ± 0.13	−2.51 ± 0.02
Contig[3515]	GH994781.1	uncharacterized protein LOC101737697 (*Bombyx mori*)	3.96 × 10^−18^	XP_004931375	−3.18 ± 0.28	−2.37 ± 0.08
Contig[5386]	GH996141.1	tetraspanin 42Ee (*Papilio xuthus*)	2.52 × 10^−32^	BAM19943	2.34 ± 0.10	2.04 ± 0.02
ECB-C-04_H06	GH992916.1	tetraspanin D107 (*Plutella xylostella*)	4.23 × 10^−75^	BAD52262	4.16 ± 0.19	2.99 ± 0.04
Contig[4527]	GH989618.1	cytochrome b561 domain-containing protein 1-like (*Bombyx mori*)	1.58 × 10^−17^	XP_004928254	−15.05 ± 1.90	−5.28 ± 1.18
Contig[4714]	GH990109.1	X box binding protein-1 (*Papilio xuthus*)	3.94 × 10^−21^	BAM18272	2.73 ± 0.13	2.28 ± 0.08
Contig[5050]	GH993665.1	salivary secreted peptide-like (*Bombyx mori*)	9.36 × 10^−10^	XP_004925327	2.94 ± 0.16	4.92 ± 0.13
Contig[5119]	GH997971.1	similar to CG3625 CG3625-PB isoform 2 (*Tribolium* *castaneum*)	7.17 × 10^−58^	EFA08684	−2.63 ± 0.09	−2.44 ± 0.08
Contig[5143]	GH995296.1	CTL-like protein 2-like, partial (*Bombyx mori*)	3.51 × 10^−49^	XP_004928594	2.26 ± 0.06	2.17 ± 0.03
Contig[5228]	GH988628.1	putative C1A cysteine protease precursor (*Manduca* *sexta*)	1.21 × 10^−130^	ADN19567	2.31 ± 0.09	3.42 ± 0.18
Contig[5707]	GH988628.1	myosin light polypeptide 9 isoform B (*Bombyx* *mori*)	3.54 × 10^−91^	NP_001103768	2.28 ± 0.08	2.53 ± 0.04
Contig[5715]	GH995679.1	putative dsRNase (*Danaus plexippus*)	1.68 × 10^−151^	EHJ64029	−3.7 ± 0.28	−2.54 ± 0.23
Contig[5729]	GH990820.1	SEC14-like protein 2-like (*Bombyx mori*)	6.23 × 10^−78^	XP_004930377	−4.23 ± 0.21	−2.79 ± 0.23
Contig[5837]	GH992838.1	unknown unsecreted protein (*Papilio xuthus*)	6.81 × 10^−15^	BAM18795	2.54 ± 0.08	3.09 ± 0.07
Contig[5872]	GH990179.1	suppressor of profilin 2 (*Papilio polytes*)	7.76 × 10^−125^	BAM20384	2.73 ± 0.04	2.20 ± 0.05
Contig[5929]	GH990692.1	unknown (*Picea* *sitchensis*)	3.96 × 10^−5^	ABK24774	3.98 ± 0.23	2.45 ± 0.09
ECB-01_G02	GH997211.1	putative actin-related protein 2/3 complex subunit 2 (*Papilio xuthus*)	4 × 10^−141^	KPI99849.1	2.45 ± 0.08	2.10 ± 0.06
ECB-02_H03	GH997311.1	saposin-like protein (*Bombyx* *mori*)	4.48 × 10^−102^	ADU03994	2.84 ± 0.46	2.87 ± 0.30
ECB-11_E02	GH997992.1	lipoyltransferase 1, mitochondrial-like isoform X1 (*Bombyx mori*)	5.91 × 10^−7^	XP_004930070	−3.47 ± 0.13	−2.34 ± 0.01
ECB-11_E06	GH997996.1	peroxisomal membrane protein 11C-like (*Bombyx mori*)	2.71 × 10^−18^	XP_004925254	−5.13 ± 0.42	−2.69 ± 0.12
ECB-14_B03	GH998214.1	ubiquitin conjugating enzyme E2 (*Danaus plexippus*)	3.08 × 10^−125^	EHJ71699	2.54 ± 0.10	2.72 ± 0.06
ECB2_C08	GH996766.1	farnesyl diphosphate synthase (*Bombyx* *mori*)	1.76 × 10^−26^	BAF62113	−2.96 ± 0.28	−2.64 ± 0.04
ECB-23_E01	GH999038.1	alpha-tocopherol transfer protein-like (*Bombus* *terrestris*)	1.57 × 10^−30^	XP_003494152	3.63 ± 0.04	2.88 ± 0.12
ECB-26_F05	GH999293.1	spaghetti squash (*Papilio xuthus*)	9.83 × 10^−4^	BAM20106	2.26 ± 0.18	2.57 ± 0.05
ECB-C-05_C05	GH992946.1	lysine-specific demethylase 6A-like (*Bombyx mori*)	1.41 × 10^−5^	XP_004932357	6.49 ± 0.60	5.50 ± 0.94
ECB-C-06_B02	GH993009.1	hypothetical protein KGM_13045 (*Danaus plexippus*)	3.22 × 10^−15^	EHJ67514	−3.50 ± 0.17	−2.68 ± 0.14
ECB-C-11_A06	GH993413.1	hypothetical protein KGM_21983 (*Danaus plexippus*)	9.75 × 10^−4^	EHJ65944	−4.22 ± 0.87	−3.00 ± 0.13
ECB-V-05_D03	GH994574.1	hypothetical protein KGM_08118 (*Danaus plexippus*)	1.09 × 10^−43^	EHJ71129	3.25 ± 0.16	2.68 ± 0.07
ECB-V-07_C07	GH994728.1	hypothetical protein KGM_13882 (*Danaus plexippus*)	5.67 × 10^−10^	EHJ75224	3.37 ± 0.03	2.19 ± 0.06
ECB-V-07_D03	GH994735.1	similar to CG6040 (*Papilio polytes*)	2.69 × 10^−34^	BAM19302	6.60 ± 0.67	6.08 ± 0.06
ECB-V-14_C12	GH995289.1	vacuolar protein sorting 37B (*Danaus plexippus*)	8.09 × 10^−64^	EHJ74800	2.30 ± 0.03	2.82 ± 0.06
ECB-V-23_C10	GH996010.1	putative growth hormone regulated TBC protein 1 (*Danaus plexippus*)	8.68 × 10^−88^	EHJ70386	2.27 ± 0.06	2.10 ± 0.02
ECB-V-25_C10	GH996179.1	hypothetical protein CAEBREN_00117 (*Caenorhabditis brenneri*)	6.84 × 10^−7^	EGT31568	5.91 ± 0.44	6.27 ± 0.19
ECB-V-25_F09	GH996208.1	presqualene diphosphate phosphatase-like (*Bombyx mori*)	2.97 × 10^−42^	XP_004934264	3.46 ± 0.17	3.21 ± 0.02
gi_133905779	EL928629.1	vanin-like protein 1 precursor (*Papilio xuthus*)	5.08 × 10^−16^	BAM18114	−9.20 ± 1.07	−5.67 ± 0.21
gi_133906199	EL929039.1	hypothetical protein KGM_15512 (*Danaus plexippus*)	1.13 × 10^−7^	EHJ79041	3.17 ± 0.36	2.58 ± 0.14
gi_133906419	EL929259.1	lipophorin receptor protein (*Spodoptera litura*)	8.96 × 10^−5^	ADN04911	2.64 ± 0.03	2.44 ± 0.03
J-ECB-06_D11	GH991264.1	aquaporin (*Bombyx* *mori*)	8.49 × 10^−69^	AFC34081	−9.45 ± 1.07	−3.20 ± 0.09
J-ECB-17_A10	GH991305.1	ras-related protein Rab-18-like (*Bombyx mori*)	7.05 × 10^−46^	XP_004926852	2.42 ± 0.20	2.36 ± 0.20
J-ECB-21_G07	GH989088.1	leucine-rich repeat-containing protein 70-like (*Bombyx mori*)	2.34 × 10^−26^	XP_004930073	2.56 ± 0.08	2.33 ± 0.06
J-ECB-29_G03	GH992468.1	IST1 homolog (*Bombyx mori*)	5.86 × 10^−69^	XP_004931988	5.72 ± 0.41	6.66 ± 0.33
J-ECB-33_G10	GH989292.1	transmembrane protein 205-like (*Bombyx mori*)	1.29 × 10^−26^	XP_004923868	2.66 ± 0.20	3.41 ± 0.12
J-ECB-35_F06	GH990233.1	heme oxygenase (*Bombyx* *mori*)	6.66 × 10^−48^	NP_001040361	2.29 ± 0.10	2.22 ± 0.03
J-ECB-38_B03	GH991444.1	inhibitor of growth protein 3-like (*Bombyx mori*)	2.09 × 10^−33^	XP_004931798	2.75 ± 0.17	2.06 ± 0.02
J-ECB-39_H09	GH992207.1	extracellular domains-containing protein CG31004-like isoform X1 (*Bombyx mori*)	4.65 × 10^−70^	XP_004925418	3.36 ± 0.20	2.67 ± 0.05
J-ECB-41_A03	GH988071.1	ankyrin-2-like (*Bombyx mori*)	1.06 × 10^−17^	XP_004926186	3.23 ± 0.41	3.66 ± 3.47
J-ECB-43_F12	GH989190.1	EF-hand domain-containing protein CG10641-like (*Bombyx mori*)	4.24 × 10^−70^	XP_004931797	8.91 ± 0.44	7.79 ± 0.11
J-ECB-47_A02	GH990851.1	hepatocyte growth factor-regulated tyrosine kinase substrate-like (*Bombyx mori*)	3.71 × 10^−39^	XP_004932480	3.77 ± 0.01	3.78 ± 0.25
J-ECB-49_H10	GH992172.1	cystathionine γ-lyase (*Bombyx mori*)	1.28 × 10^−19^	NP_001040113	6.29 ± 0.33	2.97 ± 0.08

* S:Plant and R:Plant denote S- and R-strain larvae, respectively, fed transgenic corn leaves expressing Cry1Ab. **#** Each contig sequence has multiple GenBank EST ID, but the listed ID represents the EST of longest sequence deposited in GenBank.

**Table 3:** Sequences of primers used in RT-qPCR analysis.

Putative Gene Name	EST ID	GenBank EST ID	Primer Sequences 5′–3′	Strain
Chymotrypsin-like	Contig[0130]	GH999462	TCGGGACAACTGGTCTAGCCGCACTCGTCGTTAGGTATC	S
Chymotrypsin-like	Contig[0147]	GH998267	GCTGGTTCCCTCTACTGGTCGAGATGGTGTTGGAGAAGGC	S
Trypsin	ECB-C-18-B11	GH994018	CACAAAGTCCTGGAGGAAGATTCGTTCACGCCTGTCTGTTGC	S
Chymotrypsin-like	Contig[4021]	GH987247	ACCTGCCTACCAGCGTTTCCCGAAGCCTGAAGCAATAGC	S
Chymotrypsin-like	Contig[0573]	GH999314	TCAGTGGAACCCGTGGAACCAGTGCGATTGGTTGGATGG	S
Trypsin-like	Contig[3466]	GH997407	GAGTGGGGTCTTCCTTCAGGCAGCAATGTCGTTGTTAAGCG	S
Trypsin-like	Contig[3118]	GH990367	AACTACGACGGAGAAAAGGCACTGCTCATTATCAATA	S
Trypsin-like	Contig[0293]	GH997809	CTCGTAGAAGAAGATGATGTCGTTAGAGTCTTCGTTA	S
Serine protease	Contig[2151]	GH996088	GTAAGACTGGTCGGTGGTAAAGTCGGCTCCAAGAACACAATG	S
Aminopeptidase N	ECB-V-02-D07	GH994338	AACTTACCTTCTGGCTATTCTGGCTATAACTTCGTA	S
Aminopeptidase N	ECB-V-05-D12	GH994609	GTCAACGAAATTGTCATCAGTCATATTCTGGCTGTA	S
Chymotrypsin-like	Contig[1519]	GH999020	CGAACTTATCCAATGACATTCACTTGGTTGATTTGTG	R
Trypsin	Contig[0113]	GH998291	TTCTACTGTGAACATCCTTCCAAAGATTCAAATCCC	R
Aminopeptidase N	J-ECB-41_F04	GH988241	AAGGACTAACTGCTATGACTGCCAAGTTGATTCTTA	R
Trypsin-like	Contig[0578]	GH996481	CATCCGAGATTCTCTTATGCAGTGTTATTGTAGAACTCT	S and R
Trypsin-like	Contig[0039]	GH997464	ATGCGTACCTTCATCGTTCTACGCCATCTCAGGGTATTGGTTAATG	S and R
Trypsin precursor	Contig[0243]	GH998064	GCCAGCATTACACCTTCCGTCGCAGTTCTCGTAGTAAGAC	S and R
Aminopeptidase N	Contig[1398]	GH9910376	TCTGTAGTCTGGTTCACATTATCCACTCACCTCCGCTGTATCC	S and R
Aminopeptidase N	Contig[4776]	GH998970	TTCCAAACACATTTTCTTGAAGCGTATTGTCCTCTAT	S and R
Aminopeptidase N	Contig[5112]	GH997440	CTTCAACAGCCCACTGGAGAGACGCAAGACATATTAGGTAACAGC	S and R

## Supplementary Materials

Supplementary materials can be found at www.mdpi.com/1422-0067/18/2/301/s1.
