# Supplementary material for: Comparisons of Transcriptional Profiles of Gut Genes between Cry1Ab-Resistant and Susceptible Strains of Ostrinia nubilalis Revealed Genes Possibly Related to the Adaptation of Resistant Larvae to Transgenic Cry1Ab Corn
Source: Int J Mol Sci. 2017 Jan 30;18(2):301. doi: 10.3390/ijms18020301 (PMC5343837; doi:10.3390/ijms18020301)
Supplement: Supplementary file 1 [file ijms-18-00301-s001.pdf]

# Supplementary Materials: Comparisons of Transcriptional Profiles of Gut Genes between Cry1Ab-Resistant and Susceptible Strains of *Ostrinia nubilalis* Revealed Genes Possibly Related to the Adaptation of Resistant Larvae to Transgenic Cry1Ab Corn

**Jianxiu Yao, Yu-Cheng Zhu, Nanyan Lu, Lawrent L. Buschman and Kun Yan Zhu**

**Table S1.** The complete list of gut genes in R- and S-strain larvae of *O. nubilalis* with significantly differential expression after fed transgenic Cry1Ab corn leaves as compared with those fed non-transgenic corn (control) leaves.

| EST ID | GenBank EST ID # | *E*-Value | Sequence Description | Expression Ratio * | |
|---|---|---|---|---|---|
| | | | | S:Cry1Ab Corn | R:Cry1Ab Corn |
| **Trypsin, chymotrypsin** | | | | | |
| Contig[0039] | GH997464.1 | 0.0 | trypsin serine protease (*Ostrinia furnacalis*) | −3.63 | −2.01 |
| Contig[0113] | GH998291 | $2.45 \times 10^{-69}$ | trypsin (*Helicoverpa armigera*) | | −4.67 |
| Contig[0130] | GH999462.1 | $1.07 \times 10^{-88}$ | chymotrypsin-like protease C3 (*Heliothis virescens*) | −2.95 | |
| Contig[0147] | GH998279.1 | $7.20 \times 10^{-104}$ | chymotrypsin (*Helicoverpa armigera*) | −2.28 | |
| Contig[0243] | GH998064.1 | $1 \times 10^{-174}$ | trypsin-like serine protease 12 (*Ostrinia nubilalis*) | −9.02 | −5.89 |
| Contig[2151] | GH996088.1 | $1.26 \times 10^{-39}$ | larval chymotrypsin-like protein precursor (*Aedes aegypti*) | −3.71 | |
| Contig[0293] | GH997809 | $7.89 \times 10^{-71}$ | trypsin-like serine proteinase t26 | −3.22 | |
| Contig[0573] | GH999314.1 | $3.39 \times 10^{-84}$ | chymotrypsin-like serine protease | −2.47 | |
| Contig[0578] | GH996481.1 | $4.69 \times 10^{-100}$ | trypsin-like serine proteinase t26 | −4.68 | −2.52 |
| Contig[1519] | GH999020.1 | $8.46 \times 10^{-84}$ | chymotrypsin-like protease | | −2.26 |
| Contig[3118] | GH990367 | $4.65 \times 10^{-57}$ | trypsin-like serine proteinase t26 | −3.05 | |
| Contig[3466] | GH997407 | $1.69 \times 10^{-55}$ | trypsin-like serine protease | −2.88 | |
| ECB-C-18_B11 | GH994018 | $7.13 \times 10^{-114}$ | silk gland derived serine protease | −3.00 | |
| Contig[4021] | GH987247.1 | $1.03 \times 10^{-109}$ | chymotrypsin-like serine protease | −2.57 | |
| ECB-C-13_A03 | GH993568.1 | $5.60 \times 10^{-7}$ | chymotrypsin-C-like (*Meleagris gallopavo*) | | −2.30 |
| J-ECB-52_A02 | GH987844.1 | $3.93 \times 10^{-8}$ | serine protease inhibitor 3 (*Tabanus yao*) | 10.71 | |
| J-ECB-08_D11 | GH992096.1 | $3.97 \times 10^{-27}$ | putative protease inhibitor 4 (*Lonomia obliqua*) | 19.48 | |
| Contig[1913] | GH997417.1 | $4.00 \times 10^{-120}$ | serpin-2 (*Bombyx mandarina*) | 2.13 | |
| **aminopeptidase** | | | | | |
| Contig[1398] | GH993761.1 | 0.0 | aminopeptidase N | −2.32 | 2.05 |
| Contig[4776] | GH998970.1 | 0.0 | aminopeptidase N | 2.59 | 2.31 |
| Contig[5112] | GH997440.1 | 0.0 | aminopeptidase N | −2.90 | −2.32 |
| ECB-V-02_D07 | GH994338 | $2.20 \times 10^{-99}$ | aminopeptidase N | −3.50 | |

| | | | | | |
|---|---|---|---|---|---|
| ECB-V-05_D12 | GH994609 | $2.03 \times 10^{-112}$ | aminopeptidase N | −3.28 | |
| J-ECB-41_F04 | GH988241 | $2.06 \times 10^{-61}$ | aminopeptidase N | | 2.09 |
| **carboxylesterase and carboxypeptidase** | | | | | |
| Contig[0266] | GH999150.1 | $2.63 \times 10^{-170}$ | carboxylesterase-11 | −2.61 | |
| Contig[0115] | GH997328.1 | $2.13 \times 10^{-80}$ | carboxylesterase (*Loxostege sticticalis*) | 25.18 | |
| Contig[1179] | GH995965.1 | $1.22 \times 10^{-75}$ | carboxylesterase | | −2.84 |
| Contig[3820] | GH999448.1 | $1.80 \times 10^{-99}$ | carboxylesterase | −2.67 | |
| Contig[4729] | GH992299.1 | $4.59 \times 10^{-70}$ | carboxylesterase | −17.96 | |
| Contig[5372] | GH998289.1 | $1.90 \times 10^{-98}$ | carboxylesterase-11 | −2.69 | |
| Contig[5691] | GH990344 .1 | $6 \times 10^{-56}$ | carboxyl/choline esterase (*Helicoverpa* armigera) | −10.91 | −4.57 |
| ECB-V-21_D08 | GH995856.1 | $1.14 \times 10^{-21}$ | carboxylesterase | −4.55 | |
| J-ECB-07_G03 | GH991809.1 | $1.95 \times 10^{-7}$ | carboxylesterase | 16.37 | |
| J-ECB-09_D02 | GH992373.1 | $4.32 \times 10^{-105}$ | carboxylesterase (*Spodoptera litura*) | −2.86 | |
| Contig[5791] | EL929195.1 | $3.92 \times 10^{-39}$ | carboxyl/choline esterase CCE021b (*Helicoverpa armigera*) | | −2.87 |
| ECB-27_F04 | GH999378.1 | $3.26 \times 10^{-86}$ | carboxyl choline esterase cce006b | −2.71 | |
| Contig[2666] | GH990614.1 | $2.47 \times 10^{-83}$ | carboxypeptidase A | | −2.82 |
| Contig[3400] | GH997499.1 | $5.15 \times 10^{-52}$ | zinc carboxypeptidase A 1 | −2.79 | |
| Contig[3637] | GH997603.1 | $5.71 \times 10^{-75}$ | carboxypeptidase C (*Culicoides sonorensis*) | −2.35 | |
| Contig[3784] | GH997291.1 | $4.64 \times 10^{-76}$ | midgut carboxypeptidase (*Loxostege sticticalis*) | −2.21 | |
| ECB-V-29_E10 | GH996536.1 | $3 \times 10^{-20}$ | midgut carboxypeptidase 2 (*Danaus plexippus*) | −17.64 | −4.19 |
| J-ECB-42_B07 | GH988427.1 | $6.87 \times 10^{-40}$ | zinc carboxypeptidase A 1 (*Culex quinquefasciatus*) | −2.37 | |
| Contig[0077] | GH998660.1 | $1.94 \times 10^{-165}$ | caboxypeptidase 4 (*Mamestra configurata*) | −2.12 | |
| Contig[0009] | GH992549.1 | $8 \times 10^{-78}$ | zinc carboxypeptidase A 1 (*Danaus plexippus*) | −3.85 | −2.09 |
| Contig[0019] | GH998697.1 | $3 \times 10^{-106}$ | plasma glutamate carboxypeptidase (*Danaus plexippus*) | −6.65 | −2.28 |
| Contig[3603] | GH993634.1 | $1.37 \times 10^{-115}$ | cathepsin b | 2.98 | |
| **Chitin related transcripts** | | | | | |
| Contig[0505] | GH998325.1 | 0.0 | chitin binding PM protein (*Helicoverpa armigera*) | −3.3 | −2.43 |
| Contig[0188] | GH997506.1 | 0.0 | chitinase (*Ostrinia nubilalis*) | −6.00 | |
| Contig[4654] | GH996835.1 | $2.28 \times 10^{-65}$ | peritrophic membrane chitin binding protein (*Loxostege sticticalis*) | −2.39 | |
| Contig[0233] | GH989367.1 | $1.25 \times 10^{-66}$ | chitin deacetylase 2 (*Mamestra brassicae*) | −2.60 | −2.44 |
| ECB-C-05_D05 | GH992955.1 | $6.25 \times 10^{-21}$ | glucosamine-fructose-6-phosphate aminotransferase 2 (*Culex quinquefasciatus*) | −2.88 | −2.10 |
| Contig[4598] | GH998948.1 | $4.98 \times 10^{-95}$ | glucosamine-6-phosphate *N*-acetyltransferase (*Bombyx mori*) | | 2.26 |
| **Signal transduction** | | | | | |
| Contig[3760] | GH992475.1 | $5.95 \times 10^{-96}$ | small GTP-binding protein (*Bombyx mori*) | 2.24 | |
| ECB-14_B10 | GH998220.1 | $2.17 \times 10^{-16}$ | protein kinase c inhibitor (*Bombyx mori*) | | 3.48 |
| J-ECB-37_F07 | GH991243.1 | $1.50 \times 10^{-56}$ | receptor for activated protein kinase C (*Helicoverpa armigera*) | 2.76 | |

| | | | | | |
|---|---|---|---|---|---|
| J-ECB-33_E10 | GH989160.1 | $4.58 \times 10^{-51}$ | ATP-binding cassette sub-family B member 1 (*Trichoplusia ni*) | 2.59 | |
| J-ECB-10_B09 | GH988438.1 | $4.06 \times 10^{-22}$ | Oxidative stress-induced growth inhibitor 1 (*Camponotus floridanus*) | −2.36 | |
| ECB-V-27_D10 | GH996357.1 | $9.61 \times 10^{-64}$ | Hypoxia up-regulated protein 1 (*Acromyrmex echinatior*) | | −2.59 |
| Contig[5194] | GH993661.1 | $8.21 \times 10^{-79}$ | signal sequence receptor (*Biston betularia*) | | −2.18 |
| ECB-30_C02 | GH999600.1 | $4.24 \times 10^{-79}$ | signal sequence receptor β subunit (*Bombyx mori*) | | −2.35 |
| **Transcription regulator factors** | | | | | |
| Contig[5330] | GH990640.1 | $2.39 \times 10^{-54}$ | Rho GTPase activating protein, putative (*Pediculus humanus corporis*) | | 2.94 |
| Contig[3833] | GH993952.1 | $8.94 \times 10^{-20}$ | DNA-binding nuclear protein p8 (*Simulium guianense*) | | 3.78 |
| ECB-09_F07 | GH997837.1 | $1.68 \times 10^{-65}$ | Rho GTPase-activating protein 12-like (*Acyrthosiphon pisum*) | 2.70 | 2.76 |
| ECB-V-14_D07 | GH995295.1 | $5 \times 10^{-11}$ | Reverse transcriptase (*Ostrinia nubilalis*) | 2.30 | 2.22 |
| **Heat shock protein** | | | | | |
| Contig[0227] | GH997898.1 | 0.0 | heat shock protein 70 | 2.36 | 2.06 |
| Contig[2669] | GH990174.1 | $6.38 \times 10^{-99}$ | heat shock cognate 70 protein | | −2.65 |
| gi_133905913 | EL928755 | $1.21 \times 10^{-17}$ | heat shock cognate 70 protein (*Loxostege sticticalis*) | | −2.97 |
| J-ECB-29_F11 | GH992461.1 | $5.73 \times 10^{-75}$ | heat shock protein hsp23.7 (*Bombyx mori*) | | −14.3 |
| **Transporter** | | | | | |
| Contig[0814] | GH998546.1 | $1 \times 10^{-27}$ | sodium-bile acid cotransporter | −14.14 | −4.21 |
| Contig[1314] | GH993616.1 | $2 \times 10^{-73}$ | putative amino acid transporter (*Danaus plexippus*) | −3.80 | −2.52 |
| Contig[4763] | GH998142.1 | $2 \times 10^{-65}$ | sodium-bile acid cotransporter | −10.83 | −6.56 |
| Contig[5496] | GH998547.1 | $2 \times 10^{-103}$ | sugar transporter | −2.66 | −2.36 |
| Contig[5743] | GH993678.1 | $7 \times 10^{-97}$ | sodium shloride dependent amino acid transporter | −3.96 | −2.55 |
| ECB-16_F07 | GH998441.1 | $4.45 \times 10^{-57}$ | sodium-dependent phosphate transporter | −5.51 | 2.83 |
| ECB-21_C09 | GH998857.1 | $6.29 \times 10^{-30}$ | sugar transporter | −4.90 | −2.12 |
| ECB-22_H11 | GH998995.1 | $1.20 \times 10^{-78}$ | Sodium- and chloride-dependent glycine transporter 2 (*Harpegnathos saltator*) | | −2.45 |
| ECB-V-21_D11 | GH995859.1 | $1.47 \times 10^{-26}$ | monocarboxylate transporter | | −2.21 |
| ECB-V-22_E06 | GH995942.1 | $1.46 \times 10^{-83}$ | GDP-fucose transporter, putative (*Nasonia vitripennis*) | 2.02 | 2.39 |
| gi_133906576 | EL929414 | $3.68 \times 10^{-30}$ | ATP-binding cassette transporter subfamily B (*Bombyx mori*) | | −2.39 |
| J-ECB-39_E12 | GH992066.1 | $3.41 \times 10^{-15}$ | sugar transporter (*Anopheles darlingi*) | −9.56 | −4.34 |
| J-ECB-52_H10 | GH988032.1 | $3.05 \times 10^{-17}$ | monocarboxylate transporter | −3.30 | −2.13 |
| J-ECB-42_F09 | GH988676.1 | $3.01 \times 10^{-7}$ | zinc transporter foi-like isoform 2 (*Apis mellifera*) | | 6.84 |
| J-ECB-55_E04 | GH988996.1 | $6.00 \times 10^{-38}$ | monocarboxylate transporter | −3.62 | −3.88 |
| Contig[3828] | GH993919.1 | $2.85 \times 10^{-22}$ | transport protein Sec61 γ subunit (*Bombyx mori*) | | −2.07 |
| **Xenobiotics detoxification enzyme** | | | | | |
| Contig[4722] | GH997947.1 | $1.40 \times 10^{-87}$ | ctyochrome P450 CYP6AB4 (*Bombyx mandarina*) | −2.93 | |
| Contig[5056] | GH995380.1 | $7.10 \times 10^{-25}$ | cytochrome P450 | | 2.14 |

| | | | | | |
|---|---|---|---|---|---|
| Contig[5080] | GH996933.1 | $9.88 \times 10^{-95}$ | cytochrome P450 monooxygenase cyp4m5 | | 3.84 |
| ECB-C-03_D08 | GH992802.1 | $3 \times 10^{-106}$ | cytochrome P450 (*Spodoptera litura*) | | 2.62 |
| J-ECB-10_F11 | GH988695.1 | $3.50 \times 10^{-50}$ | cytochrome P450 | 2.38 | |
| J-ECB-21_B02 | GH988756.1 | $2.52 \times 10^{-99}$ | cytochrome P450 | −3.34 | |
| Contig[0004] | GH992504.1 | $4.79 \times 10^{-52}$ | glutathione *S*-transferase (*Choristoneura fumiferana*) | −8.76 | −2.95 |
| Contig[0012] | GH987677.1 | $5.36 \times 10^{-54}$ | microsomal glutathione transferase (*Heliothis virescens*) | | −2.59 |
| **Antibacterial related proteins** | | | | | |
| gi_133905829 | EL928679 | $1.82 \times 10^{-13}$ | hinnavin II (*Pieris rapae*) | 11.84 | |
| Contig[5720] | GH993782.1 | $1.08 \times 10^{-93}$ | immune-related Hdd13 (*Hyphantria cunea*) | −2.41 | |
| Contig[4668] | GH989903.1 | $5.75 \times 10^{-63}$ | peptidoglycan recognition protein | | −2.84 |
| J-ECB-60_D07 | GH987186.1 | $1.88 \times 10^{-24}$ | antibacterial protein (*Heliothis virescens*) | | 3.49 |
| **Other metabolic enzymes** | | | | | |
| BM2_D02 | GH992543.1 | $6.85 \times 10^{-60}$ | lipase | −2.90 | |
| Contig[0016] | GH997476.1 | $1.25 \times 10^{-84}$ | lipase 1 precursor | −2.56 | |
| Contig[0029] | GH998728.1 | $2.12 \times 10^{-104}$ | gastric lipase-like protein (*Epiphyas postvittana*) | −5.44 | |
| Contig[0140] | GH998810.1 | $7 \times 10^{-136}$ | pancreatic lipase-related protein 2-like (*Bombyx mori*) | | −11.58 |
| Contig[0450] | GH997309.1 | $1.24 \times 10^{-121}$ | lipase | −4.71 | |
| Contig[0923] | GH999509.1 | $7.84 \times 10^{-80}$ | lipase | −7.96 | −10.45 |
| Contig[1081] | GH998825.1 | $6.98 \times 10^{-55}$ | pancreatic lipase-like protein (*Epiphyas postvittana*) | −4.83 | −5.02 |
| Contig[2857] | GH998380.1 | $2.58 \times 10^{-124}$ | lipase | −3.19 | |
| Contig[4980] | GH998599.1 | $1.22 \times 10^{-55}$ | lipase 1 precursor | | −2.69 |
| Contig[5664] | GH997481.1 | $1.40 \times 10^{-47}$ | lipase | −4.33 | |
| ECB-07_C04 | GH997675.1 | $4.43 \times 10^{-23}$ | Lipase 1 (*Camponotus floridanus*) | −4.64 | |
| J-ECB-56_D02 | GH989456.1 | $2.80 \times 10^{-21}$ | Gastric triacylglycerol lipase (*Camponotus floridanus*) | −4.57 | |
| Contig[1486] | GH997709.1 | $1.25 \times 10^{-63}$ | c-5 sterol desaturase erg32-like (*Bombyx mori*) | −7.75 | −2.79 |
| Contig[1897] | GH997709.1 | $9.20 \times 10^{-92}$ | c-5 sterol desaturase erg32-like (*Bombyx mori*) | −7.03 | −2.13 |
| ECB-V-05_B10 | GH994560.1 | $7.96 \times 10^{-116}$ | acyl-CoA-Δ9-3a-desaturase (*Dendrolimus punctatus*) | | −2.19 |
| ECB-C-20_B10 | GH994173.1 | $1.50 \times 10^{-68}$ | carbohydrate kinase-like (*Tribolium castaneum*) | −2.37 | |
| J-ECB-61_C03 | GH987254.1 | $7.52 \times 10^{-47}$ | UDP-glucosyltransferase (*Bombyx mori*) | 2.57 | 2.08 |
| ECB-V-19_F07 | GH995711.1 | $1.06 \times 10^{-52}$ | UDP-glucosyltransferase (*Bombyx mori*) | 2.87 | |
| J-ECB-30_H09 | GH988109.1 | $1 \times 10^{-120}$ | Myrosinase 1 (*Papilio xuthus*) | −4.16 | |
| ECB-C-14_E07 | GH993705.1 | $2.07 \times 10^{-77}$ | β-glucosidase precursor (*Spodoptera frugiperda*) | −5.25 | |
| J-ECB-15_G11 | GH990690.1 | $3.69 \times 10^{-43}$ | putative 6-phosphofructo-2-kinase/fructose-2,6-biphosphatase 4 (*Danaus plexippus*) | 2.65 | 2.42 |
| Contig[0075] | GH997236.1 | $5.55 \times 10^{-143}$ | glycoside hydrolases | −3.44 | |
| Contig[0700] | GH998843.1 | $2.36 \times 10^{-121}$ | sucrose-6-phosphate hydrolase | −2.98 | |
| Contig[0911] | GH999492.1 | $3.62 \times 10^{-153}$ | juvenile hormone epoxide hydrolase | −2.39 | |
| Contig[4515] | GH987646.1 | $2.23 \times 10^{-70}$ | γ-glutamyl hydrolase | −2.56 | −2.49 |

| | | | | | |
|---|---|---|---|---|---|
| Contig[4896] | GH998937.1 | $3 \times 10^{-134}$ | epoxide hydrolase 3-like (*Plutella xylostella*) | −2.65 | |
| Contig[5232] | GH987506.1 | $8.08 \times 10^{-57}$ | glycoside hydrolases | −29.36 | |
| Contig[4425] | GH988573.1 | $4.78 \times 10^{-105}$ | enolase | −2.36 | |
| J-ECB-46_D03 | GH990544.1 | $4.07 \times 10^{-54}$ | ubiquitin carboxyl-terminal hydrolase 14-like isoform 1 (*Bombus terrestris*) | 2.42 | |
| J-ECB-33_G12 | GH989302.1 | $1.24 \times 10^{-95}$ | juvenile hormone epoxide hydrolase-like protein 1 (*Bombyx mori*) | −6.20 | |
| J-ECB-50_D10 | GH987676.1 | $9.67 \times 10^{-85}$ | glycoside hydrolases | −5.16 | |
| J-ECB-58_H02 | GH990678.1 | $1.85 \times 10^{-115}$ | platelet-activating factor acetyl-hydrolase isoform 1b $\alpha$ subunit (*Aedes aegypti*) | 2.16 | |
| BM2_B12 | GH992538.1 | $2.23 \times 10^{-48}$ | hydroxysteroid (17-β) dehydrogenase 8 | −2.24 | −2.30 |
| Contig[0022] | GH992601.1 | $2.89 \times 10^{-53}$ | alcohol dehydrogenase | −6.41 | −2.36 |
| Contig[0030] | GH997149.1 | 0.0 | aldehyde dehydrogenase, mitochondrial | −2.88 | |
| Contig[0231] | GH997486.1 | $3.12 \times 10^{-64}$ | short-chain dehydrogenase | | −3.25 |
| Contig[1778] | GH994839.1 | $1.56 \times 10^{-57}$ | acyl-CoA dehydrogenase | −2.50 | −2.63 |
| Contig[3532] | GH997374.1 | 0.0 | Isocitrate dehydrogenase (NADP), mitochondrial (*Papilio xuthus*) | −3.13 | |
| Contig[3814] | GH994966.1 | $2.32 \times 10^{-112}$ | alcohol dehydrogenase (*Bombyx mori*) | −3.07 | |
| Contig[4310] | GH997290.1 | $1.82 \times 10^{-56}$ | 3-hydroxybutyrate dehydrogenase type 2 | −2.11 | |
| Contig[5289] | GH990630.1 | $9.57 \times 10^{-26}$ | isovaleryl coenzyme A dehydrogenase (*Heliothis virescens*) | −5.68 | |
| ECB-01_G12 | GH997221.1 | $4.02 \times 10^{-146}$ | zinc-containing alcohol dehydrogenase (*Bombyx mori*) | | 2.17 |
| ECB-14_D05 | GH998238.1 | $8.51 \times 10^{-63}$ | aldehyde dehydrogenase (*Heliothis virescens*) | −3.09 | |
| gi_133906638 | EL929475 | $8.18 \times 10^{-30}$ | retinol dehydrogenase (*Heliothis virescens*) | −10.53 | −10.95 |
| Contig[0362] | GH997850.1 | $2.85 \times 10^{-89}$ | carbonyl reductase | −4.82 | −2.14 |
| Contig[1032] | GH999460.1 | $5.61 \times 10^{-15}$ | $\gamma$-interferon inducible lysosomal thiol reductase (*Glossina morsitans*) | −2.48 | |
| Contig[2754] | GH997997.1 | $2.30 \times 10^{-79}$ | carbonyl reductase | 2.51 | |
| Contig[4521] | GH989023.1 | $8.07 \times 10^{-98}$ | aldo-keto reductase | | −27.07 |
| Contig[5655] | GH996386.1 | $3.83 \times 10^{-79}$ | lysosomal thiol reductase IP30 isoform 2 (*Bombyx mori*) | | −2.29 |
| **Others** | | | | | |
| Contig[0308] | GH997849.1 | $1.26 \times 10^{-21}$ | peritrophin type-A domain protein 2 (*Mamestra configurata*) | | −5.72 |
| Contig[0218] | GH998427.1 | $1.48 \times 10^{-117}$ | NADPH cytochrome b5 reductase (*Spodoptera exigua*) | −2.14 | −2.02 |
| Contig[5933] | GH997219.1 | $2.23 \times 10^{-07}$ | Abhydrolase domain-containing protein 4 (*Harpegnathos saltator*) | 2.38 | |
| Contig[3484] | GH987848.1 | $3.95 \times 10^{-54}$ | mitochondrial cytochrome c (*Bombyx mori*) | | −2.65 |
| Contig[1432] | GH994698.1 | $5.19 \times 10^{-133}$ | catalase (*Bombyx mori*) | −2.36 | |
| Contig[1310] | GH993550.1 | $1.51 \times 10^{-78}$ | uridine phosphorylase (*Aedes aegypti*) | −2.30 | |
| Contig[5679] | GH988679.1 | $3.47 \times 10^{-81}$ | phosphoserine aminotransferase | −4.08 | −3.26 |

| | | | | | |
|---|---|---|---|---|---|
| Contig[0098] | EL929505.1 | $2.72 \times 10^{-6}$ | JHBP-like protein (*Diploptera punctata*) | −3.76 | |
| Contig[0165] | GH998248.1 | $1.66 \times 10^{-78}$ | actin depolymerizing factor | 2.26 | |
| Contig[0241] | GH997622.1 | $6.80 \times 10^{-12}$ | conserved hypothetical protein (*Culex quinquefasciatus*) | 2.16 | |
| Contig[0382] | GH998342.1 | 0.0 | β-1,3-glucanase (*Helicoverpa armigera*) | −2.21 | |
| Contig[0407] | GH997662.1 | $3.18 \times 10^{-31}$ | sensory appendage protein 3 (*Manduca sexta*) | | −57.87 |
| Contig[0535] | GH990867.1 | $4.20 \times 10^{-8}$ | hypothetical protein AaeL_AAEL009036 (*Aedes aegypti*) | −2.71 | −2.53 |
| Contig[0810] | GH999215.1 | $2.25 \times 10^{-150}$ | calreticulin (*Galleria mellonella*) | | −3.37 |
| Contig[0827] | GH996906.1 | $2.72 \times 10^{-21}$ | Ankyrin repeat domain protein Wolbachia endosymbiont of (*Culex quinquefasciatus*) | −3.26 | |
| Contig[0924] | GH999510.1 | $4.70 \times 10^{-46}$ | carbonic anhydrase (*Aedes aegypti*) | −2.12 | |
| Contig[0976] | GH987290.1 | $1.92 \times 10^{-120}$ | ferritin (*Manduca sexta*) | −2.33 | |
| Contig[1250] | GH992423.1 | $3.90 \times 10^{-44}$ | aquaporin | −2.47 | |
| Contig[1305] | GH998434.1 | 0.0 | angiotensin converting enzyme (*Spodoptera littoralis*) | −2.19 | |
| Contig[1573] | GH997739.1 | $9.41 \times 10^{-14}$ | hypothetical conserved protein | 2.27 | 2.76 |
| Contig[1640] | GH991677.1 | $7.33 \times 10^{-26}$ | fatty acid-binding protein-like (*Bombus terrestris*) | −2.76 | |
| Contig[1868] | GH995418.1 | $1.16 \times 10^{-43}$ | ER protein reticulon (*Aedes aegypti*) | 2.74 | 2.21 |
| Contig[1880] | GH995131.1 | $1.46 \times 10^{-6}$ | adipokinetic 3 (*Helicoverpa armigera*) | 5.46 | 4.09 |
| Contig[1953] | GH997798.1 | $3 \times 10^{-145}$ | prostaglandin reductase 1-like (*Papilio xuthus*) | −2.71 | −2.51 |
| Contig[2576] | GH992555.1 | $5 \times 10^{-91}$ | hypothetical protein g.11100 (*Pectinophora gossypiella*) | −3.03 | |
| Contig[2896] | GH987914.1 | $4.35 \times 10^{-58}$ | lipid storage droplets surface binding protein 2 | 2.98 | |
| Contig[2947] | GH992278.1 | $3.52 \times 10^{-52}$ | high-affinity copper uptake protein | −5.88 | |
| Contig[3004] | GH990119.1 | $2.66 \times 10^{-89}$ | β lactamase domain | −5.38 | |
| Contig[3035] | GH997788.1 | $7.67 \times 10^{-47}$ | anterior fat body protein | −12.82 | |
| Contig[3237] | GH992782.1 | $5.35 \times 10^{-86}$ | dipeptidyl peptidase 4 | −2.74 | |
| Contig[3266] | GH987418.1 | $2 \times 10^{-6}$ | hypothetical protein RR48_06180 (*Papilio machaon*) | −3.02 | |
| Contig[3372] | GH987678.1 | $2.75 \times 10^{-15}$ | macrophage migration inhibitory factor | 4.26 | |
| Contig[3454] | GH987480.1 | $4.99 \times 10^{-15}$ | α1,3-fucosyltransferase C (*Apis mellifera*) | 2.30 | |
| Contig[3515] | GH994781.1 | $3.96 \times 10^{-18}$ | uncharacterized protein LOC101737697 (*Bombyx mori*) | −3.17 | −2.37 |
| Contig[3585] | GH988106.1 | $7.56 \times 10^{-91}$ | glutathione peroxidase | 2.14 | |
| Contig[3619] | GH992525.1 | $2.24 \times 10^{-6}$ | takeout/JHBP-like protein (*Diploptera punctata*) | −3.65 | |
| Contig[3674] | GH998445.1 | $5.16 \times 10^{-60}$ | bcr-associated protein, bap (*Aedes aegypti*) | 3.15 | |
| Contig[3708] | GH991311.1 | $4.46 \times 10^{-95}$ | muscle protein 20-like protein (*Papilio* xuthus) | 2.15 | |
| Contig[3740] | GH988393.1 | $2.25 \times 10^{-89}$ | FK506-binding protein (*Bombyx mori*) | | −3.83 |
| Contig[5365] | GH993317.1 | $6.23 \times 10^{-69}$ | tetraspanin 2A, isoform A (*Drosophila melanogaster*) | 2.22 | |
| Contig[5386] | GH996141.1 | $2.52 \times 10^{-32}$ | tetraspanin E118 (*Bombyx mori*) | 2.34 | 2.04 |
| ECB-C-04_H06 | GH992916.1 | $4.23 \times 10^{-75}$ | tetraspanin d107 | 4.16 | 2.99 |
| Contig[3869] | GH997175.1 | $4.22 \times 10^{-49}$ | creg1 precursor(Cellular repressor of E1A-stimulated genes 1) (*Tribolium castaneum*) | | −2.18 |

| | | | | | |
|---|---|---|---|---|---|
| Contig[4000] | GH997153.1 | $1.31 \times 10^{-59}$ | ryanodine receptor-like protein (*Phlebotomus papatasi*) | 2.28 | |
| Contig[4287] | GH991765.1 | $3.21 \times 10^{-125}$ | β-tubulin (*Bombyx mori*) | 2.56 | |
| Contig[4527] | GH989618.1 | $1.58 \times 10^{-17}$ | conserved hypothetical protein (*Culex quinquefasciatus*) | −15.05 | −5.28 |
| Contig[4714] | GH990109.1 | $3.94 \times 10^{-21}$ | similar to X box binding protein-1 CG9415-PA (*Tribolium castaneum*) | 2.73 | 2.28 |
| Contig[4784] | GH992658.1 | $1.55 \times 10^{-60}$ | leucine repeat-rich protein (*Heliconius melpomene melpomene*) | | 2.83 |
| Contig[4916] | GH990108.1 | $4.27 \times 10^{-16}$ | hypothetical protein TcasGA2_TC002334 (*Tribolium castaneum*) | | 3.60 |
| Contig[5038] | GH991382.1 | $2.82 \times 10^{-20}$ | MBF2 (*Samia cynthia*) | | 2.39 |
| Contig[5045] | GH998223.1 | $5.41 \times 10^{-84}$ | ubiquitin-conjugating enzyme rad6 (*Aedes aegypti*) | 2.02 | |
| Contig[5050] | GH993665.1 | $9.36 \times 10^{-10}$ | salivary secreted peptide-like (*Bombyx mori*) | 2.94 | 4.92 |
| Contig[5114] | GH997529.1 | $4.37 \times 10^{-100}$ | xaa-pro dipeptidase peptidase (Aedes *aegypti*) | −2.08 | |
| Contig[5119] | GH997971.1 | $7.17 \times 10^{-58}$ | similar to CG3625 CG3625-PB isoform 2 (*Tribolium castaneum*) | −2.63 | −3.78 |
| Contig[5123] | GH998229.1 | $1.19 \times 10^{-69}$ | Abl interactor 2 (*Harpegnathos saltator*) | | 2.41 |
| Contig[5133] | GH999457.1 | $9.52 \times 10^{-78}$ | myosin light chain 2 (*Antheraea pernyi*) | 2.98 | |
| Contig[5136] | GH992820.1 | $2.79 \times 10^{-101}$ | ceramidase (*Aedes aegypti*) | −2.90 | |
| Contig[5143] | GH995296.1 | $3.51 \times 10^{-49}$ | ctl2 (*Aedes aegypti*) | 2.25 | 2.17 |
| Contig[5148] | GH992405.1 | $7.10 \times 10^{-42}$ | Coronin-2B (*Harpegnathos saltator*) | 2.79 | |
| Contig[5168] | GH989763.1 | $4.13 \times 10^{-124}$ | tropomyosin-1 (*Bombyx mori*) | 3.64 | |
| Contig[5228] | GH988628.1 | $1.21 \times 10^{-130}$ | putative C1A cysteine protease precursor (*Manduca sexta*) | 2.31 | 3.42 |
| Contig[5259] | GH997723.1 | $6.74 \times 10^{-40}$ | dipeptidyl-peptidase (*Aedes aegypti*) | −3.10 | |
| Contig[5301] | GH998943.1 | $1.45 \times 10^{-48}$ | insect intestinal mucin 3 (*Helicoverpa armigera*) | −2.40 | |
| Contig[5397] | GH991289.1 | $9.12 \times 10^{-61}$ | astacin (*Mamestra configurata*) | −4.06 | |
| Contig[5632] | GH989110.1 | $1.98 \times 10^{-9}$ | GH18999 (*Drosophila grimshawi*) | | 5.40 |
| Contig[5707] | GH994232.1 | $3.54 \times 10^{-91}$ | myosin light polypeptide 9 isoform B (*Bombyx mori*) | 2.28 | 2.53 |
| Contig[5715] | GH997945.1 | $1.68 \times 10^{-151}$ | alkaline nuclease (*Bombyx mori*) | −3.70 | −2.54 |
| Contig[5729] | GH990820.1 | $6.31 \times 10^{-78}$ | SEC14-like protein 2-like (*Apis mellifera*) | −4.23 | −2.79 |
| Contig[5744] | GH997656.1 | $1.27 \times 10^{-06}$ | 363_100_1 protein (*Mamestra* configurata) | | −2.67 |
| Contig[5800] | GH991870.1 | $1.26 \times 10^{-16}$ | larval cuticle protein 14 (*Manduca sexta*) | 3.14 | |
| Contig[5826] | GH999419.1 | $7.07 \times 10^{-68}$ | troponin I (*Loxostege sticticalis*) | 2.57 | |
| Contig[5837] | GH992838.1 | $6.81 \times 10^{-15}$ | unknown unsecreted protein (*Papilio xuthus*) | 2.54 | 3.09 |
| Contig[5838] | GH993157.1 | $2.23 \times 10^{-18}$ | canopy-1-like (*Apis mellifera*) | | −2.83 |
| Contig[5872] | GH990179.1 | $7.76 \times 10^{-125}$ | suppressor of profilin 2 (*Papilio polytes*) | 2.73 | 2.20 |
| Contig[5929] | GH990692.1 | $3.96 \times 10^{-5}$ | unknown (*Picea sitchensis*) | 3.98 | 2.45 |
| Contig[5975] | GH996882.1 | $4.78 \times 10^{-5}$ | SocE (*Bacillus cereus* W) | | −2.94 |
| Contig[6042] | GH996189.1 | $2.64 \times 10^{-36}$ | chemosensory protein (*Papilio xuthus*) | 6.71 | |
| ECB-01_G02 | GH997211.1 | $4 \times 10^{-141}$ | putative actin-related protein 2/3 complex subunit 2 (*Papilio xuthus*) | 2.45 | 2.10 |
| ECB-02_H03 | GH997311.1 | $4.48 \times 10^{-102}$ | saposin-like protein (*Bombyx mori*) | 2.84 | 2.87 |

| | | | | | |
|---|---|---|---|---|---|
| ECB-03_E12 | GH997370.1 | $4.71 \times 10^{-64}$ | DUF233 protein (*Heliothis virescens*) | 9.52 | |
| ECB-11_E02 | GH997992.1 | $5.91 \times 10^{-7}$ | lipoyltransferase 1, mitochondrial-like isoform X1 (*Bombyx mori*) | −3.47 | −2.34 |
| ECB-11_E06 | GH997996.1 | $2.71 \times 10^{-18}$ | peroxisomal membrane protein 11C-like (*Bombyx mori*) | −5.13 | −2.69 |
| ECB-12_D10 | GH998071.1 | $1.38 \times 10^{-48}$ | putative WD repeat domain 13 (Wdr13) (*Heliconius melpomene*) | | 2.27 |
| ECB-14_B03 | GH998214.1 | $3.08 \times 10^{-125}$ | ubiquitin conjugating enzyme E2 (*Danaus plexippus*) | 2.54 | 2.72 |
| ECB-15_C06 | GH998316.1 | $8.43 \times 10^{-74}$ | translocon-associated protein γ isoform 2 (*Bombyx mori*) | | −2.54 |
| ECB-16_E08 | GH998430.1 | $3.12 \times 10^{-49}$ | mitochondrial aminotransferase (*Camponotus floridanus*) | | −2.94 |
| ECB-17_C10 | GH998499.1 | $2.35 \times 10^{-56}$ | protein disulfide isomerase (*Helicoverpa armigera*) | | −2.60 |
| ECB-18_C11 | GH998591.1 | $3.21 \times 10^{-152}$ | β-tubulin (*Bombyx mori*) | 3.74 | |
| ECB-18_E05 | GH998608.1 | $1.32 \times 10^{-113}$ | thioredoxin (*Bombyx mori*) | 2.35 | |
| ECB-19_A07 | GH998655.1 | $6.22 \times 10^{-69}$ | astacin (*Mamestra configurata*) | −3.34 | |
| ECB2_C08 | GH996766.1 | $1.76 \times 10^{-26}$ | farnesyl diphosphate synthase (*Bombyx mori*) | −2.96 | −2.64 |
| ECB-21_E01 | GH998872.1 | $8.95 \times 10^{-13}$ | CG12926-PA-like protein (*Helicoverpa armigera*) | −2.48 | |
| ECB-23_E01 | GH999038.1 | $1.57 \times 10^{-30}$ | α-tocopherol transfer protein-like (*Bombus terrestris*) | 3.63 | 2.87 |
| ECB-25_G02 | GH999219.1 | $3.13 \times 10^{-38}$ | CG3862-PA-like protein (*Plutella xylostella*) | 2.09 | |
| ECB-26_F04 | GH999292.1 | $2.98 \times 10^{-74}$ | Arp2/3 complex p21 subunit (*Spodoptera frugiperda*) | 2.42 | |
| ECB-26_F05 | GH999293.1 | $9.83 \times 10^{-4}$ | spaghetti squash (*Papilio xuthus*) | 2.26 | 2.57 |
| ECB-27_B09 | GH999338.1 | $5.92 \times 10^{-33}$ | ankyrin repeat domain protein | −2.86 | |
| ECB-C-02_F10 | GH992743.1 | $2.71 \times 10^{-108}$ | Rab32 (*Helicoverpa armigera*) | 2.45 | |
| ECB-C-03_G05 | GH992827.1 | $9.31 \times 10^{-17}$ | conserved hypothetical protein (*Culex quinquefasciatus*) | 3.32 | |
| ECB-C-04_A08 | GH992850.1 | $3.23 \times 10^{-18}$ | β-1 tubulin (*Spodoptera frugiperda*) | 3.33 | |
| ECB-C-05_C05 | GH992946.1 | $1.41 \times 10^{-5}$ | lysine-specific demethylase 6A-like (*Bombyx mori*) | 6.49 | 5.50 |
| ECB-C-06_B02 | GH993009.1 | $3.22 \times 10^{-15}$ | hypothetical protein KGM_13045 (*Danaus plexippus*) | −3.50 | −2.68 |
| ECB-C-11_A06 | GH993413.1 | $9.75 \times 10^{-4}$ | hypothetical protein KGM_21983 (*Danaus plexippus*) | −4.22 | −3.00 |
| ECB-C-12_E09 | GH993531.1 | $7.65 \times 10^{-33}$ | kinesin-associated protein, putative (*Aedes aegypti*) | 2.14 | |
| ECB-C-13_F05 | GH993625.1 | $1.91 \times 10^{-79}$ | heterogeneous nuclear ribonucleoprotein A1 (*Bombyx mori*) | 2.28 | |
| ECB-C-14_B06 | GH993671.1 | $1.54 \times 10^{-6}$ | glycolipid transfer protein (*Bombyx mori*) | | 2.55 |
| ECB-C-14_D01 | GH993689.1 | $1.12 \times 10^{-5}$ | hypothetical protein AND_12479 (*Anopheles darlingi*) | −2.85 | |
| ECB-C-17_F04 | GH993969.1 | $2.37 \times 10^{-53}$ | rrm-containing protein seb-4, putative (*Pediculus humanus corporis*) | −2.12 | |
| ECB-C-20_C09 | GH994183.1 | $5.77 \times 10^{-20}$ | conserved hypothetical protein (*Culex quinquefasciatus*) | 2.26 | |
| ECB-V-02_E10 | GH994349.1 | $3.62 \times 10^{-60}$ | scavenger mRNA-decapping enzyme DcpS-like isoform 1 (*Apis mellifera*) | 2.17 | |
| ECB-V-05_D03 | GH994574.1 | $1.09 \times 10^{-43}$ | Adhesion-regulating molecule 1 precursor (*Pediculus humanus corporis*) | 3.25 | 2.68 |
| ECB-V-05_G12 | GH994609.1 | $5.33 \times 10^{-45}$ | ecdysteroid UDP-glucosyltransferase | 3.54 | |
| ECB-V-07_C07 | GH994728.1 | $5.67 \times 10^{-10}$ | hypothetical protein KGM_13882 (*Danaus plexippus*) | 3.37 | 2.19 |

| | | | | | |
|---|---|---|---|---|---|
| ECB-V-07_D03 | GH994735.1 | $2.69 \times 10^{-34}$ | similar to CG6040 (*Papilio polytes*) | 6.60 | 6.08 |
| ECB-V-08_G01 | GH994850.1 | $2.44 \times 10^{-66}$ | conserved hypothetical protein (*Culex quinquefasciatus*) | 2.07 | |
| ECB-V-08_G03 | GH994852.1 | $3.61 \times 10^{-61}$ | ecdysteroid UDP-glucosyltransferase | 3.17 | |
| ECB-V-09_A03 | GH994876.1 | $5.51 \times 10^{-85}$ | Mps one binder kinase activator-like 1 (*Harpegnathos saltator*) | 2.84 | |
| ECB-V-12_E11 | GH995156.1 | $2.43 \times 10^{-49}$ | Dipeptidyl peptidase 4 (*Harpegnathos saltator*) | −3.57 | |
| ECB-V-14_C12 | GH995289.1 | $8.09 \times 10^{-64}$ | vacuolar protein sorting 37b | 2.30 | 2.82 |
| ECB-V-15_B06 | GH995352.1 | $4.97 \times 10^{-6}$ | visgun, isoform A (*Drosophila melanogaster*) | 3.52 | |
| ECB-V-15_E06 | GH995381.1 | $8.36 \times 10^{-19}$ | Receptor expression-enhancing protein 1 (*Harpegnathos saltator*) | 2.18 | |
| ECB-V-18_A08 | GH995588.1 | $1.09 \times 10^{-6}$ | fatty acid binding protein | | −2.79 |
| ECB-V-19_E04 | GH995700.1 | $3.15 \times 10^{-42}$ | sialic acid synthase-like (*Xenopus tropicalis*) | | 2.16 |
| ECB-V-22_G04 | GH995962.1 | $1.42 \times 10^{-9}$ | CG42837 (*Drosophila melanogaster*) | −3.14 | |
| ECB-V-23_A09 | GH995988.1 | $3.96 \times 10^{-21}$ | fatty acid transport protein (*Ostrinia scapulalis*) | −2.41 | |
| ECB-V-23_C10 | GH996010.1 | $8.68 \times 10^{-88}$ | putative growth hormone regulated TBC protein 1 (*Danaus plexippus*) | 2.27 | 2.10 |
| ECB-V-23_H01 | GH996053.1 | $3.19 \times 10^{-42}$ | WW domain binding protein wBP-2 (*Glossina morsitans morsitans*) | 2.42 | |
| ECB-V-25_C10 | GH996179.1 | $6.84 \times 10^{-7}$ | hypothetical protein CAEBREN_00117 (*Caenorhabditis brenneri*) | 5.91 | 6.27 |
| ECB-V-25_F09 | GH996208.1 | $2.97 \times 10^{-42}$ | presqualene diphosphate phosphatase-like (*Bombyx mori*) | 3.46 | 3.21 |
| ECB-V-26_H03 | GH996306.1 | $1.59 \times 10^{-16}$ | AMP dependent CoA ligase (*Aedes aegypti*) | −5.28 | |
| ECB-V-29_B10 | GH996506.1 | $4.72 \times 10^{-57}$ | farnesyltransferase/geranylgeranyltransferase type I $\alpha$ subunit (*Bombyx mori*) | 2.28 | |
| ECB-V-29_G10 | GH996557.1 | $1.35 \times 10^{-72}$ | acyl-CoA oxidase (*Heliothis virescens*) | | −2.42 |
| gi_133905779 | EL928629 | $5.08 \times 10^{-16}$ | vanin-like protein 1 | −9.20 | −5.67 |
| gi_133906199 | EL929039 | $1.13 \times 10^{-7}$ | hypothetical protein KGM_15512 (*Danaus plexippus*) | 3.17 | 2.58 |
| gi_133906407 | EL929247 | $8.35 \times 10^{-35}$ | kynureninase (*Bombyx mori*) | | −3.30 |
| gi_133906419 | EL929259 | $8.96 \times 10^{-5}$ | lipophorin receptor protein (*Spodoptera litura*) | 2.63 | 2.44 |
| gi_133906904 | EL929734 | $1.57 \times 10^{-57}$ | GK17133 (*Drosophila willistoni*) | 2.12 | |
| gi_133906913 | EL929743 | $3.03 \times 10^{-5}$ | similar to CG3823 CG3823-PA (*Tribolium castaneum*) | 4.37 | |
| J-ECB-04_E11 | GH990310.1 | $7.43 \times 10^{-77}$ | sorting nexin (*Culex quinquefasciatus*) | | 3.06 |
| J-ECB-05_E12 | GH990826.1 | $9.99 \times 10^{-34}$ | actin 3 isoform, putative (*Tribolium castaneum*) | | 2.38 |
| J-ECB-06_D11 | GH991264.1 | $8.49 \times 10^{-69}$ | aquaporin (*Bombyx mori*) | −9.45 | −3.20 |
| J-ECB-08_B02 | GH991953.1 | $2.43 \times 10^{-31}$ | sensory appendage protein 3 (*Manduca sexta*) | | −71.78 |
| J-ECB-11_C08 | GH988987.1 | $5.76 \times 10^{-75}$ | ADP ribosylation factor-like protein (*Phlebotomus papatasi*) | 2.14 | |
| J-ECB-14_D04 | GH989986.1 | $7.87 \times 10^{-19}$ | CG2765 CG2765-PA (*Tribolium castaneum*) | | 2.49 |
| J-ECB-14_H06 | GH990255.1 | $4.44 \times 10^{-4}$ | similar to CG14661-PA (*Apis mellifera*) | 6.28 | |
| J-ECB-17_A10 | GH991305.1 | $7.05 \times 10^{-46}$ | ras-related protein Rab-18-like (*Bombyx mori*) | 2.42 | 2.36 |
| J-ECB-21_F05 | GH989017.1 | $1.27 \times 10^{-12}$ | Kunitz-type protease inhibitor precursor (*Galleria mellonella*) | | −4.66 |
| J-ECB-21_G07 | GH989088.1 | $2.34 \times 10^{-26}$ | leucine-rich repeat-containing protein 70-like (*Bombyx mori*) | 2.55 | 2.33 |

| | | | | | |
|---|---|---|---|---|---|
| J-ECB-24_F10 | GH990510.1 | $1.68 \times 10^{-71}$ | p94-like protein | | 3.83 |
| J-ECB-24_G10 | GH990570.1 | $1.75 \times 10^{-32}$ | putative midgut protein (*Phlebotomus perniciosus*) | 4.16 | |
| J-ECB-25_A10 | GH990720.1 | $7.12 \times 10^{-37}$ | 2-acylglycerol *O*-acyltransferase 1 (*Harpegnathos saltator*) | −2.23 | |
| J-ECB-25_D01 | GH990842.1 | $1.34 \times 10^{-43}$ | myelin proteolipid (*Biston betularia*) | 2.29 | |
| J-ECB-29_G03 | GH992468.1 | $5.86 \times 10^{-69}$ | IST1 homolog (*Bombyx mori*) | 5.72 | 6.66 |
| J-ECB-29_H09 | GH992490.1 | $1.19 \times 10^{-49}$ | Inhibitor of growth protein 3 (*Camponotus floridanus*) | 2.59 | |
| J-ECB-33_D08 | GH989092.1 | $3.78 \times 10^{-104}$ | pyridoxine 5′-phosphate oxidase (*Bombyx mori*) | −2.38 | |
| J-ECB-33_G10 | GH989292.1 | $1.29 \times 10^{-26}$ | transmembrane protein 205-like (*Bombyx mori*) | 2.66 | 3.41 |
| J-ECB-35_F06 | GH990233.1 | $6.66 \times 10^{-48}$ | heme oxygenase (*Bombyx mori*) | 2.29 | 2.22 |
| J-ECB-37_E05 | GH991174.1 | $1.10 \times 10^{-43}$ | oxidoreductase (*Acromyrmex echinatior*) | −2.77 | |
| J-ECB-38_B03 | GH991444.1 | $2.09 \times 10^{-33}$ | inhibitor of growth protein 3 | 2.75 | 2.06 |
| J-ECB-39_F07 | GH992097.1 | $1.35 \times 10^{-69}$ | cytidylate kinase | −2.21 | |
| J-ECB-39_H09 | GH992207.1 | $4.65 \times 10^{-70}$ | extracellular domains-containing protein CG31004-like isoform X1 (*Bombyx mori*) | 3.36 | 2.67 |
| J-ECB-40_D07 | GH987842.1 | $3.45 \times 10^{-73}$ | troponin C (*Bombyx mori*) | 2.22 | |
| J-ECB-41_A03 | GH988071.1 | $1.06 \times 10^{-17}$ | ankyrin-2-like (*Bombyx mori*) | 3.23 | 3.65 |
| J-ECB-41_H07 | GH988338.1 | $1.46 \times 10^{-81}$ | G10 protein (*Bombyx mori*) | | 2.05 |
| J-ECB-42_A08 | GH988386.1 | $2.15 \times 10^{-21}$ | RGS-GAIP interacting protein GIPC (*Bombyx mori*) | | 2.24 |
| J-ECB-42_C06 | GH988476.1 | $9.42 \times 10^{-25}$ | glucosylceramidase-like (*Monodelphis domestica*) | −3.45 | |
| J-ECB-43_F12 | GH989190.1 | $4.24 \times 10^{-70}$ | EF-hand domain-containing protein CG10641-like (*Bombyx mori*) | 8.91 | 7.79 |
| J-ECB-47_A02 | GH990851.1 | $3.71 \times 10^{-39}$ | hepatocyte growth factor-regulated tyrosine kinase substrate-like (*Bombyx mori*) | 3.77 | 3.78 |
| J-ECB-49_C04 | GH991887.1 | $1.10 \times 10^{-110}$ | actin (*Spodoptera exigua*) | 2.18 | |
| J-ECB-49_H10 | GH992172.1 | $1.28 \times 10^{-19}$ | cystathionine $\gamma$-lyase (*Bombyx mori*) | 6.29 | 2.97 |
| J-ECB-50_C10 | GH987666.1 | $4.80 \times 10^{-76}$ | S-adenosylmethionine decarboxylase proenzyme (*Camponotus floridanus*) | | 2.46 |
| J-ECB-50_F10 | GH987698.1 | $2.36 \times 10^{-32}$ | elongation factor-1$\alpha$ (*Corcyra cephalonica*) | −2.08 | |
| J-ECB-53_A10 | GH988057.1 | $1.07 \times 10^{-21}$ | hypothetical protein TcasGA2_TC008350 (*Tribolium castaneum*) | 2.21 | |
| J-ECB-54_F04 | GH988562.1 | $6.00 \times 10^{-25}$ | troponin T transcript variant A (*Bombyx mandarina*) | 4.03 | |
| J-ECB-58_A02 | GH990235.1 | $2.89 \times 10^{-45}$ | cuticular protein RR-1 motif 23 (*Bombyx mori*) | 19.61 | |
| J-ECB-58_A11 | GH990291.1 | $7.31 \times 10^{-104}$ | innexin 2 (*Heliothis virescens*) | | 2.49 |
| J-ECB-60_B03 | GH987159.1 | $9.42 \times 10^{-43}$ | pyroglutamyl-peptidase 1-like (*Apis mellifera*) | −2.33 | |
| J-ECB-60_C07 | GH987175.1 | $5.23 \times 10^{-43}$ | probable 4-coumarate--CoA ligase 3-like isoform 2 (*Acyrthosiphon pisum*) | −5.18 | |
| J-ECB-60_H07 | GH987229.1 | $1.45 \times 10^{-35}$ | disulfide-isomerase A6 (*Culex tarsalis*) | | −2.48 |

* S:Cry1Ab corn and R:Cry1Ab corn denote S- and R-strain larvae fed transgenic corn leaves expressing Cry1Ab toxin, respectively. # Each contig sequence has multiple Genbank EST ID and the listed ID represents the ID of the longest EST sequence in GenBank.

**Table S2.** List of 48 common differentially expressed gut genes in S-strain larvae fed artificial diet containing Cry1Ab protoxin as compared with those fed artificial diet without Cry1Ab protoxin, and S- and R-strain larvae fed transgenic corn leaves expressing Cry1Ab toxin as compared with those fed non-transgenic corn leaves.

| EST ID | GenBank EST ID # | Sequence Description | Expression Ratio * | | |
|---|---|---|---|---|---|
| | | | S:protoxin | S:Cry1Ab Corn | R:Cry1Ab Corn |
| BM2_B12 | GH992538 | hypothetical protein TcasGA2_TC011405 (*Tribolium castaneum*) | −2.07 | −2.24 | -2.30 |
| Contig[0004] | GH992504 | glutathione S-transferase (*Choristoneura fumiferana*) | −3.53 | −8.76 | −2.95 |
| Contig[0009] | GH992549 | putative carboxypeptidase A-like (*Nasonia vitripennis*) | −2.19 | −3.85 | −2.09 |
| Contig[0019] | GH998697 | glutamate carboxypeptidase-like isoform 1 (*Apis mellifera*) | −3.14 | −6.64 | −2.28 |
| Contig[0243] | GH998064 | trypsin (*Helicoverpa armigera*) | −2.45 | −9.02 | −5.89 |
| Contig[0814] | GH998546 | sodium/bile acid cotransporter (*Tribolium castaneum*) | −5.03 | −14.14 | −4.21 |
| Contig[1081] | GH998825 | pancreatic lipase-like protein (*Epiphyas postvittana*) | −2.57 | −4.83 | −5.01 |
| Contig[1314] | GH993616 | amino acid transporter (*Bombyx mori*) | −4.68 | −3.80 | −2.51 |
| Contig[1486] | GH997709 | C-5 sterol desaturase-like (*Acyrthosiphon pisum*) | −4.96 | −7.75 | −2.79 |
| Contig[1897] | GH997709 | C-5 sterol desaturase-like (*Acyrthosiphon pisum*) | −4.48 | −7.03 | −2.13 |
| Contig[1953] | GH997798 | NADP-dependent oxidoreductase (*Bombyx mori*) | −3.27 | −2.71 | −2.51 |
| Contig[4527] | GH989618 | conserved hypothetical protein (*Culex quinquefasciatus*) | −7.89 | −15.05 | −5.28 |
| Contig[4763] | GH998142 | sodium-bile acid cotransporter (*Aedes aegypti*) | −4.39 | −10.83 | −6.56 |
| Contig[5679] | GH988679 | phosphoserine aminotransferase (*Antheraea pernyi*) | −2.18 | −4.08 | −3.26 |
| Contig[5743] | GH993678 | amino acid transporter (*Bombyx mori*) | −5.40 | −3.96 | −2.55 |
| ECB-02_H03 | GH997311 | saposin-like protein (*Bombyx mori*) | −2.02 | 2.84 | 2.87 |
| ECB−14_E05 | GH998249 | n/a | −3.62 | 3.27 | 2.56 |
| ECB-C-04_H06 | GH992916 | tetraspanin D107 (*Bombyx mori*) | −2.70 | 4.16 | 2.99 |
| ECB-C-11_A06 | GH993413 | hypothetical protein EAI_08582 (*Harpegnathos saltator*) | −2.86 | −4.22 | −3.00 |
| gi_133905779 | EL928629 | Vanin-like protein 1 (*Culex quinquefasciatus*) | −3.35 | −9.20 | −5.67 |
| gi_133906638 | EL929475 | retinol dehydrogenase (*Heliothis virescens*) | −3.61 | −10.53 | −10.95 |
| J-ECB-39_E12 | GH992066 | sugar transporter (*Culex quinquefasciatus*) | −2.65 | −9.57 | −4.35 |
| J-ECB-55_E04 | GH988996 | monocarboxylate transporter (*Aedes aegypti*) | −3.4 | −3.62 | −3.88 |
| Contig[1263] | GH996374 | n/a | 4.74 | 2.32 | 2.91 |
| Contig[1573] | GH997739 | n/a | 2.07 | 2.27 | 2.76 |
| Contig[4776] | GH998970 | SXSS-APN2 (*Ostrinia furnacalis*) | 2.17 | −2.59 | 2.31 |
| Contig[5143] | GH995296 | ctl2 (*Aedes aegypti*) | 2.32 | 2.25 | 2.17 |
| Contig[5262] | GH993421 | n/a | 5.52 | 3.58 | 4.86 |
| Contig[5386] | GH996141 | tetraspanin E118 (*Bombyx mori*) | 2.33 | 2.34 | 2.04 |
| Contig[5414] | GH997359 | Ubiquitin-63E, isoform A (*Drosophila melanogaster*) | 2.73 | 2.49 | 2.04 |
| Contig[5929] | GH990692 | n/a | 2.60 | 3.98 | 2.45 |
| Contig[6000] | GH998341 | n/a | 2.14 | 2.48 | 2.82 |
| ECB-11_E06 | GH997996 | GD21009 (*Drosophila simulans*) | 2.21 | −5.13 | −2.69 |

| | | | | | |
|---|---|---|---|---|---|
| ECB-14_H12 | GH998287 | n/a | 2.13 | 5.29 | 5.16 |
| ECB-17_F12 | GH998536 | n/a | 16.41 | 24.46 | 6.87 |
| ECB-18_B07 | GH998575 | n/a | 3.99 | 2.71 | 2.44 |
| ECB-21_C09 | GH998857 | sugar transporter protein 3 (*Bombyx mori*) | 2.52 | −4.90 | −2.12 |
| ECB-C-05_D05 | GH992955 | glucosamine-fructose-6-phosphate aminotransferase 2 (*Culex quinquefasciatus*) | 2.24 | −2.88 | −2.10 |
| ECB-V-07_D03 | GH994735 | similar to CG6040 (*Papilio polytes*) | 2.66 | 6.60 | 6.08 |
| ECB-V-16_B02 | GH995433 | n/a | 2.36 | 2.35 | 2.07 |
| ECB-V-25_C10 | GH996179 | hypothetical protein (*Aedes aegypti*) | 2.29 | 5.91 | 6.27 |
| gi_133907290 | EL930112 | n/a | 4.37 | 5.14 | 3.90 |
| J-ECB-25_H03 | GH991098 | n/a | 4.21 | 6.25 | 4.84 |
| J-ECB-29_G03 | GH992468 | IST1 homolog (*Bombus terrestris*) | 2.18 | 5.72 | 6.66 |
| J-ECB-39_H09 | GH992207 | Extracellular domains-containing protein (*Acromyrmex echinatior*) | 2.15 | 3.36 | 2.67 |
| J-ECB-47_A02 | GH990851 | hepatocyte growth factor-regulated tyrosine kinase substrate (hgs) (*Tribolium castaneum*) | 3.09 | 3.77 | 3.78 |

"*" S:protoxin denotes the S-strain larvae fed artificial diet containing Cry1Ab protoxin at the $LC_{50}$ concentration; S:Cry1Ab corn and R:Cry1Ab denote the S- and R-strain larvae fed transgenic corn leaves expressing Cry1Ab, respectively. "#" Each contig sequence has multiple Genbank EST IDs and the listed ID represents the ID of the longest EST sequence in GenBank. "n/a" The sequence does not have functional descriptions in NCBI database.

**Table S3.** List of differentially expressed gut genes in S and R-strain larvae fed non-transgenic corn leaves.

| EST ID | Sequence Description | Expression Ratio ± SE (R/S) |
|---|---|---|
| **Serine protease** | | |
| Contig[2883] | chymotrypsin | −3.01 ± 0.24 |
| Contig[5043] | chymotrypsin 7 | −2.73 ± 0.16 |
| Contig[0147] | chymotrypsin-like serine protease | −2.50 ± 0.00 |
| Contig[4021] | chymotrypsin-like serine protease | −2.21 ± 0.12 |
| Contig[0573] | chymotrypsin-like serine protease | −2.44 ± 0.09 |
| Contig[1519] | chymotrypsin-like serine protease | 3.21 ± 0.31 |
| ECB-V-27_E08 | chymotrypsin-like serine protease partial | −23.76 ± 2.21 |
| Contig[0027] | serine protease | −2.69 ± 0.15 |
| Contig[3118] | serine protease 24 | −5.30 ± 0.16 |
| ECB-05_D02 | serine protease inhibitor dipetalogastin-like | 5.97 ± 0.34 |
| J-ECB-50_G05 | serine protease partial | −42.35 ± 2.30 |
| Contig[1207] | serine protease precursor | −14.06 ± 0.33 |
| Contig[5740] | trypsin β-like | −3.36 ± 0.45 |
| Contig[0344] | trypsin-like serine protease | −4.48 ± 0.42 |
| Contig[3704] | trypsin-like serine protease | −2.38 ± 0.04 |
| Contig[4768] | trypsin-like serine protease | −5.50 ± 0.16 |
| **Aminipeptidase/ABC/ALP** | | |
| ECB-V-10_H10 | aminopeptidase | 2.36 ± 0.09 |
| Contig[1398] | aminopeptidase | −2.45 ± 0.03 |
| Contig[4776] | aminopeptidase | 4.34 ± 0.08 |
| Contig[5190] | V-type proton ATPase subunit B | 2.17 ± 0.05 |
| Contig[1309] | V-type proton ATPase subunit e 2-like | 2.25 ± 0.14 |
| J-ECB-37_G01 | V-type proton ATPase subunit S1 | 2.64 ± 0.04 |
| Contig[3154] | alkaline phosphatase-like | 3.24 ± 0.21 |
| **Transporter** | | |
| Contig[5496] | Facilitated trehalose transporter Tret1 | −2.47 ± 0.21 |
| ECB-16_F07 | inorganic phosphate cotransporter | −5.78 ± 1.16 |
| ECB-V-21_D11 | monocarboxylate transporter 5 | 2.25 ± 0.07 |
| ECB-C-20_C03 | Organic cation transporter | −6.28 ± 0.33 |
| ECB-22_H11 | sodium- and chloride-dependent glycine transporter 1-like | 3.25 ± 0.18 |
| Contig[5211] | facilitated trehalose transporter Tret1-like | 4.74 ± 0.39 |
| **Detoxification or detoxification-related genes** | | |
| Contig[4729] | truncated carboxylesterase | −2.69 ± 0.10 |
| Contig[0115] | esterase CXE14 | 4.97 ± 0.29 |
| J-ECB-07_G03 | esterase CXE14 | 7.47 ± 0.82 |
| Contig[5372] | esterase FE4-like | 2.50 ± 0.15 |
| Contig[0448] | cytochrome b561 domain-containing 2 | 2.04 ± 0.01 |
| Contig[6043] | cytochrome b5-like | −2.26 ± 0.10 |
| Contig[4079] | cytochrome b-c1 complex subunit 10 | 2.23 ± 0.05 |
| J-ECB-50_C06 | cytochrome c oxidase assembly factor 4 mitochondrial | −2.30 ± 0.16 |
| J-ECB-33_D09 | cytochrome P450 | 14.02 ± 0.32 |
| ECB5_D02 | Cytochrome P450 6B4 | −4.64 ± 0.11 |
| Contig[5056] | cytochrome P450 6k1-like | −2.57 ± 0.18 |
| J-ECB-04_G10 | cytochrome P450 6k1-like | −3.83 ± 0.23 |
| Contig[4426] | cytochrome P450 CYP12A2-like | 3.32 ± 0.17 |
| ECB-V-17_G09 | cytochrome P450 CYP12A2-like | 3.36 ± 1.22 |
| Contig[2821] | NADPH cytochrome P450 reductase | 7.60 ± 0.35 |
| ECB-12_F03 | NADPH--cytochrome P450 reductase | 3.91 ± 0.29 |

| | | |
|---|---|---|
| Contig[0643] | glutathione S-transferase 1-1-like | 3.65 ± 1.30 |
| Contig[1881] | glutathione S-transferase-like | 3.05 ± 0.95 |
| **Others** | | |
| Contig[3650] | 10 kDa secreted protein | −22.50 ± 1.00 |
| ECB-C-15_C10 | 15-hydroxyprostaglandin dehydrogenase ($NAD^+$)-like | −4.46 ± 0.38 |
| J-ECB-25_A10 | 2-acylglycerol *O*-acyltransferase 2-A-like | 2.48 ± 0.14 |
| Contig[5545] | 3-oxoacyl-(acyl-carrier-) reductase -like | 3.08 ± 0.18 |
| ECB-V-26_H03 | 4-coumarate-- ligase 1-like | 3.33 ± 0.16 |
| ECB-V-22_H03 | 4-hydroxybutyrate coenzyme A transferase | 3.21 ± 0.29 |
| ECB-C-17_G01 | 5-demethoxyubiquinone mitochondrial | −2.42 ± 0.13 |
| ECB-V-27_D07 | 5-formyltetrahydrofolate cyclo-ligase | 2.52 ± 0.08 |
| Contig[5728] | AAEL004564- partial | 2.33 ± 0.07 |
| ECB-17_C04 | AAEL017413- partial | −4.74 ± 1.42 |
| Contig[0031] | acyl-$\Delta_{11}$ desaturase | 9.08 ± 0.80 |
| ECB-V-05_B10 | acyl-desaturase Z9-1 | 3.23 ± 0.37 |
| ECB-13_A01 | adipocyte plasma membrane-associated | 2.72 ± 0.31 |
| Contig[0030] | aldehyde dehydrogenase mitochondrial-like | 2.36 ± 0.06 |
| ECB-C-18_H09 | aldose reductase-like isoform X1 | 5.78 ± 0.23 |
| J-ECB-35_D11 | alkaline ceramidase | −2.12 ± 0.01 |
| Contig[0635] | alkaline C-like | 8.36 ± 0.65 |
| Contig[1659] | alkaline C-like | 2.78 ± 0.00 |
| Contig[5759] | alkaline C-like | 3.58 ± 0.17 |
| J-ECB-33_F11 | alkaline C-like | 5.53 ± 0.36 |
| J-ECB-29_C01 | $\alpha$-(1,3)-fucosyltransferase 10-like | −2.24 ± 0.09 |
| Contig[3475] | $\alpha$-(1,3)-fucosyltransferase C-like | −2.49 ± 0.08 |
| ECB-30_C09 | $\alpha$-amylase 1-like | −2.25 ± 0.17 |
| ECB-28_F03 | $\alpha$-tocopherol transfer-like | 2.06 ± 0.02 |
| ECB-27_B09 | Ankyrin repeat domain | 2.06 ± 0.06 |
| J-ECB-06_D11 | aquaporin isoform X2 | −2.54 ± 0.33 |
| Contig[3920] | armadillo repeat-containing 6 homolog | −8.85 ± 0.92 |
| Contig[0915] | arylphorin subunit $\alpha$-like | −5.00 ± 0.41 |
| Contig[2890] | asteroid log 1 | −28.20 ± 1.28 |
| J-ECB-24_E07 | ATP-binding cassette sub-family G member 1-like | −2.31 ± 0.07 |
| ECB-C-16_D12 | ATP-dependent RNA helicase DHX30 | −3.37 ± 0.24 |
| ECB-V-30_D11 | bifunctional 3 -phosphoadenosine 5 -phosphosulfate synthase | 2.71 ± 0.09 |
| Contig[2754] | carbonyl reductase (NADPH) 1-like | 2.14 ± 0.11 |
| Contig[0362] | carbonyl reductase (NADPH) 3-like | 2.66 ± 0.19 |
| ECB-V-29_E10 | carboxypeptidase B-like | −3.05 ± 0.27 |
| Contig[5228] | cathepsin L | −4.20 ± 0.21 |
| Contig[0407] | chemosensory | 67.54 ± 20.30 |
| Contig[0505] | chitin deacetylase 1 | −2.50 ± 0.49 |
| Contig[5328] | chitinase 3 | 2.86 ± 0.14 |
| J-ECB-48_H08 | cleft lip and palate transmembrane 1 homolog | 2.31 ± 0.14 |
| ECB-15_G12 | coiled-coil domain-containing 47 | −2.18 ± 0.13 |
| Contig[1382] | collagenase-like | −266.68 ± 9.01 |
| Contig[0578] | collagenase-like | −2.04 ± 0.00 |
| Contig[0245] | collagenase-like | 3.32 ± 0.08 |
| Contig[5126] | collagenase-like | 2.70 ± 0.32 |
| ECB-C-06_B02 | cueball | −2.12 ± 0.08 |
| Contig[0348] | cyclic GMP-AMP synthase-like | −2.62 ± 0.37 |
| Contig[3758] | cyclic GMP-AMP synthase-like | −6.91 ± 1.22 |

| | | |
|---|---|---|
| J-ECB-08_G07 | cyclic GMP-AMP synthase-like | −29.87 ± 9.22 |
| ECB-V-07_H04 | DALR anticodon-binding domain-containing 3 | −2.29 ± 0.24 |
| ECB-03_A08 | DEAD-box helicase Dbp80 | 2.18 ± 0.13 |
| Contig[6024] | defense 1 | 4.97 ± 0.35 |
| Contig[1719] | $\Delta_{24}$-sterol reductase-like | 3.35 ± 1.24 |
| ECB-28_E04 | diamine acetyltransferase 2-like | 2.89 ± 0.16 |
| J-ECB-42_H12 | DNA-directed RNA polymerase II subunit GRINL1A | −2.77 ± 0.15 |
| ECB-V-11_B08 | DNAJ homolog 1 | −2.15 ± 0.04 |
| Contig[3724] | dTDP-glucose 4,6-dehydratase | −2.13 ± 0.07 |
| J-ECB-01_D10 | Dynein heavy chain axonemal | 2.55 ± 0.39 |
| J-ECB-53_E04 | dynein light chain roadblock-type 2 | −4.89 ± 1.44 |
| Contig[4781] | E3 ubiquitin- ligase sina-like isoform X3 | −6.67 ± 0.73 |
| ECB-V-14_B03 | E3 ubiquitin- ligase Su(Dx) | −2.26 ± 0.13 |
| J-ECB-04_B07 | ecdysteroid 22-kinase | −2.47 ± 0.33 |
| ECB-11_F07 | ecdysteroid-regulated 16 kDa | 4.23 ± 0.49 |
| J-ECB-43_F12 | EF-hand domain-containing D2 homolog | 2.44 ± 0.11 |
| Contig[6042] | ejaculatory bulb-specific 3-like | 3.06 ± 0.15 |
| Contig[5715] | Endonuclease mitochondrial | −2.50 ± 0.26 |
| Contig[2154] | endonuclease-reverse transcriptase | −6.90 ± 4.68 |
| J-ECB-47_A03 | endonuclease-reverse transcriptase | 3.73 ± 1.45 |
| Contig[5481] | epidermal retinol dehydrogenase 2-like | 2.79 ± 0.26 |
| Contig[1610] | epididymal secretory E1-like | −2.59 ± 0.11 |
| ECB-V-19_H05 | estrogen sulfotransferase | 3.71 ± 0.15 |
| Contig[5427] | eukaryotic translation initiation factor 4E-like | −14.84 ± 0.83 |
| Contig[0615] | fas-associated death domain | −6.16 ± 0.22 |
| Contig[0347] | Fatty acid-binding 2 | −8.20 ± 0.85 |
| Contig[0566] | fatty acid-binding 2 | 4.69 ± 0.43 |
| Contig[1085] | flexible cuticle 12-like | 10.37 ± 1.15 |
| ECB-20_C07 | GATA zinc finger domain-containing 14-like | −13.84 ± 2.02 |
| Contig[1778] | glutaryl- mitochondrial | 2.33 ± 0.21 |
| ECB-05_B10 | glycerophosphodiester phosphodiesterase | −2.19 ± 0.05 |
| Contig[0111] | hatching enzyme | −2.39 ± 0.07 |
| Contig[5397] | hatching enzyme | 2.59 ± 0.08 |
| ECB-22_B05 | helicase SKI2W | 11.26 ± 8.11 |
| J-ECB-08_H05 | Hematopoietically-expressed homeobox hhex | −2.20 ± 0.07 |
| Contig[5984] | histidine triad nucleotide-binding 1 | −2.29 ± 0.19 |
| ECB-15_A08 | histidine-rich glyco -like | 3.55 ± 0.59 |
| ECB-V-03_C02 | hsc70-interacting-like | −2.38 ± 0.08 |
| Contig[5654] | hydroxylysine kinase | 3.52 ± 0.16 |
| Contig[5010] | hypothetical protein g.12871 | −2.32 ± 0.08 |
| J-ECB-12_A05 | hypothetical protein g.14421 | −3.24 ± 0.16 |
| ECB-12_A06 | hypothetical protein g.8449 | −2.41 ± 0.08 |
| Contig[5666] | hypothetical protein OBRU01_04590 | −77.68 ± 3.96 |
| J-ECB-56_E05 | hypothetical protein RR48_07032 | −2.47 ± 0.16 |
| Contig[0251] | hypothetical protein RR48_12366 | −37.90 ± 3.08 |
| Contig[1427] | hypothetical protein RR48_12366 | −7.56 ± 4.53 |
| Contig[5720] | immune-related Hdd13 | −4.57 ± 0.21 |
| J-ECB-53_B03 | integrator complex subunit 1 isoform X2 | −3.10 ± 0.46 |
| Contig[3532] | isocitrate dehydrogenase (NADP) cytoplasmic-like | 2.30 ± 0.01 |
| ECB-V-26_D03 | isopentenyl-diphosphate Δ-isomerase 1 | −4.09 ± 0.47 |
| ECB-03_E12 | juvenile hormone binding | 9.79 ± 0.56 |
| Contig[3727] | larval cuticle 1-like | 13.46 ± 1.19 |

| | | |
|---|---|---|
| Contig[5800] | larval cuticle LCP-17-like | 3.88 ± 0.35 |
| gi_133905657 | lipase 1-like | −3.64 ± 0.54 |
| J-ECB-56_D02 | lipase 1-like | −10.87 ± 0.48 |
| ECB-07_C04 | lipase 1-like | 4.09 ± 0.42 |
| Contig[4980] | lipase 3-like | −2.34 ± 0.06 |
| J-ECB-12_D03 | lipid storage droplets surface-binding 2 | 2.49 ± 0.30 |
| ECB-V-22_G04 | Lipoate ligase | 2.18 ± 0.04 |
| ECB-11_E02 | lipoyltransferase mitochondrial isoform X1 | 4.35 ± 2.12 |
| ECB-V-23_A09 | long-chain fatty acid transport 4-like | −4.33 ± 0.77 |
| Contig[0685] | lysosomal acid phosphatase-like | 3.02 ± 0.21 |
| Contig[1923] | lysozyme 1 | 2.06 ± 0.04 |
| Contig[2229] | Maltase 1 | −2.82 ± 0.44 |
| Contig[3279] | maltase A1-like | 2.58 ± 0.12 |
| Contig[0738] | membrane alanyl aminopeptidase-like | 2.75 ± 0.17 |
| ECB-C-05_H08 | Mitochondrial import inner membrane translocase subunit Tim9 | 2.16 ± 0.04 |
| J-ECB-33_E10 | multidrug resistance 1A-like | 59.13 ± 9.42 |
| J-ECB-01_C01 | multiple epidermal growth factor-like domains 10 | −4.17 ± 0.24 |
| Contig[5453] | Myophilin | 3.37 ± 0.05 |
| gi_133905748 | myosin light chain smooth muscle-like | −2.28 ± 0.18 |
| Contig[5133] | myosin regulatory light chain 2 | 2.79 ± 0.09 |
| Contig[5707] | myosin regulatory light chain sqh | −2.41 ± 0.29 |
| ECB-26_F05 | myosin regulatory light chain sqh | −3.16 ± 0.44 |
| J-ECB-30_H09 | myrosinase 1-like | −2.37 ± 0.17 |
| Contig[1061] | NADH dehydrogenase (ubiquinone) 1β subcomplex subunit mitochondrial | 2.29 ± 0.25 |
| J-ECB-01_B08 | NFX1-type zinc finger-containing 1-like | −4.85 ± 0.50 |
| ECB-19_H01 | nose resistant to fluoxetine 6-like | −4.00 ± 0.97 |
| ECB-27_E07 | nuclease HARBI1 | 2.30 ± 0.11 |
| J-ECB-10_B09 | oxidative stress-induced growth inhibitor 1-like | −2.48 ± 0.18 |
| J-ECB-24_F10 | p94 | −3.36 ± 0.53 |
| Contig[0112] | pancreatic triacylglycerol lipase-like | −5.04 ± 1.12 |
| Contig[0450] | pancreatic triacylglycerol lipase-like | −6.49 ± 0.43 |
| Contig[3472] | pancreatic triacylglycerol lipase-like | −3.42 ± 0.10 |
| Contig[2388] | peptidoglycan recognition -like | 3.09 ± 0.52 |
| Contig[2223] | peptidoglycan-recognition LB-like | −2.73 ± 0.16 |
| Contig[1391] | peptidoglycan-recognition LB-like | 10.90 ± 0.43 |
| Contig[5021] | peritrophin type-A domain 2 | −6.79 ± 0.68 |
| Contig[1195] | phosphatidylethanolamine-binding homolog-like | −10.20 ± 1.44 |
| J-ECB-55_A05 | probable 2-oxoglutarate dehydrogenase E1 component DHKTD1 mitochondrial | −6.05 ± 1.26 |
| ECB-05_H12 | probable citrate synthase mitochondrial | 2.13 ± 0.11 |
| Contig[3585] | probable phospholipid hydroperoxide glutathione peroxidase | 2.24 ± 0.08 |
| Contig[5679] | probable phosphoserine aminotransferase | −5.73 ± 0.33 |
| Contig[5038] | probable salivary secreted peptide | 5.83 ± 1.18 |
| Contig[5205] | probable salivary secreted peptide | 4.55 ± 0.13 |
| Contig[0619] | proline-rich extensin EPR1 | 2.62 ± 0.44 |
| J-ECB-08_D11 | protease inhibitor 4 | 11.51 ± 1.82 |
| ECB-10_C01 | pyridoxal kinase | 3.42 ± 0.38 |
| J-ECB-60_B03 | Pyroglutamyl-peptidase 1 | −4.84 ± 2.49 |
| J-ECB-24_C06 | regucalcin-like | −3.69 ± 0.48 |

| | | |
|---|---|---|
| Contig[3035] | regucalcin-like | 3.77 ± 0.01 |
| J-ECB-04_F11 | regucalcin-like (*Papilio xuthus*) | 3.81 ± 0.06 |
| Contig[5349] | regulation of enolase 1-like | −8.76 ± 2.90 |
| J-ECB-54_C04 | reticulocyte-binding 2 homolog a-like | −35.47 ± 1.27 |
| ECB-C-04_B10 | retrovirus-related Pol poly from transposon TNT 1-94 | 2.34 ± 0.12 |
| ECB-C-17_F04 | RNA-binding 24-B-like | −2.53 ± 0.26 |
| Contig[0105] | RNA-directed DNA polymerase from transposon partial | 2.33 ± 0.14 |
| ECB-05_E11 | RNMT-activating mini | 3.06 ± 0.04 |
| J-ECB-07_H02 | RPII140-upstream gene | 2.47 ± 0.01 |
| ECB-C-20_B10 | sedoheptulokinase-like isoform X2 | −2.20 ± 0.12 |
| Contig[4387] | seleno K-like | −2.29 ± 0.11 |
| J-ECB-24_D12 | selT | −2.85 ± 0.10 |
| Contig[0813] | SET and MYND domain-containing 4-like | −3.35 ± 0.15 |
| ECB-V-22_F04 | SET and MYND domain-containing 4-like | −3.89 ± 0.18 |
| Contig[5961] | short-chain dehydrogenease reductase | −3.36 ± 0.17 |
| ECB-C-11_A06 | Signal CUB and EGF-like domain-containing 2 | 3.05 ± 0.53 |
| Contig[0068] | single domain major allergen | −4.45 ± 0.53 |
| Contig[4763] | solute carrier family 10 member 6-like | 3.07 ± 0.24 |
| ECB-V-07_H05 | solute carrier family riboflavin member 3-A-like | 14.64 ± 0.77 |
| Contig[6015] | SUMO-conjugating enzyme UBC9-B | −2.16 ± 0.00 |
| Contig[3086] | superoxide dismutase (Cu-Zn] | −7.44 ± 2.87 |
| Contig[6056] | synaptic vesicle glyco 2C-like | −10.37 ± 0.40 |
| gi_133906571 | testin | −2.60 ± 0.34 |
| Contig[0408] | tetraspanin E118 | −3.64 ± 0.54 |
| Contig[0010] | thioredoxin-2 | 2.16 ± 0.01 |
| Contig[4717] | Threonine dehydratase | 2.65 ± 0.29 |
| J-ECB-41_A03 | transient receptor potential channel pyrexia-like isoform X2 | −5.09 ± 1.08 |
| ECB-C-15_H04 | Translation elongation factor 2 | 14.76 ± 3.62 |
| ECB-28_F07 | transmembrane 42 | −2.38 ± 0.13 |
| J-ECB-40_D07 | troponin C | 2.14 ± 0.14 |
| J-ECB-54_F04 | troponin T | 3.14 ± 0.28 |
| ECB-C-04_A08 | tubulin β chain | 2.68 ± 0.09 |
| ECB-18_C11 | tubulin β-1 chain | 2.22 ± 0.09 |
| Contig[5247] | Tudor domain-containing 1 | −2.99 ± 0.42 |
| J-ECB-33_E12 | U11 U12 small nuclear ribonucleo 25 kDa | 2.55 ± 0.15 |
| ECB-V-29_H03 | ubiquitin carboxyl-terminal hydrolase 46 | −2.63 ± 0.10 |
| ECB-V-05_G12 | UDP-glucuronosyltransferase 2B15-like | 6.47 ± 0.68 |
| Contig[5837] | uncharacterized LOC106121242 | −2.50 ± 0.12 |
| Contig[4263] | uncharacterized oxidoreductase TM_0325-like | 2.38 ± 0.07 |
| Contig[5802] | uncharacterized protein LOC105842367 isoform X2 (*Bombyx mori*) | −5.67 ± 0.79 |
| J-ECB-11_E05 | uridine 5 -monophosphate synthase | 2.43 ± 0.08 |
| gi_133905779 | vanin 2 isoform X2 | 4.14 ± 0.25 |
| ECB-V-12_E11 | venom dipeptidyl peptidase 4-like isoform X1 | −2.52 ± 0.08 |
| Contig[2624] | YIF1B | −7.17 ± 0.72 |
| ECB-C-05_F02 | zinc finger CCHC domain-containing 24-like | −68.49 ± 7.89 |
| ECB-03_F06 | zinc finger CCHC domain-containing partial | −5.34 ± 0.50 |
| J-ECB-15_E12 | zinc finger CCHC domain-containing partial | −39.00 ± 3.57 |

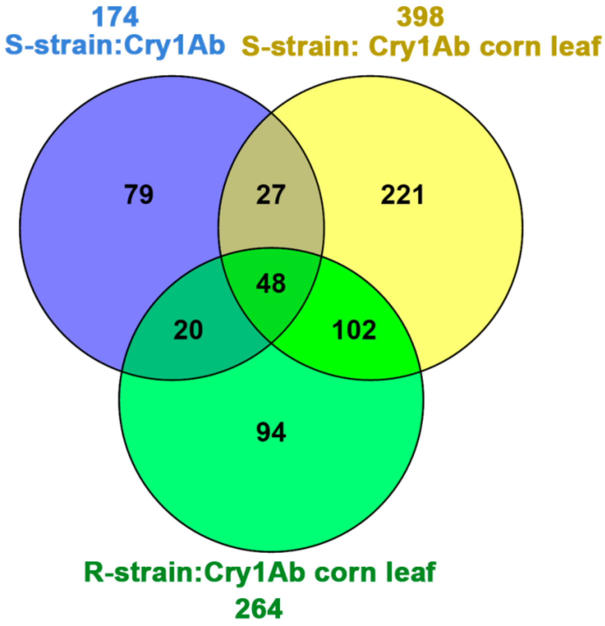


**Figure S1.** A Venn diagram showing the number of differentially expressed gut genes in S- and R-strain larvae of *O. nubilalis* fed transgenic corn leaves expressing Cry1Ab toxin and in S-strain larvae fed artificial diet containing Cry 1Ab protoxin. S-strain:Cry1Ab (slate blue) denotes S-strain larvae fed artificial diet containing Cry1Ab protoxin ($LC_{50}$) (18], whereas S-strain:Cry1Ab corn leaf (yellow) and R-strain:Cry1Ab corn leaf (green) denote S- and R-strain larvae fed transgenic corn leaves expressing Cry1Ab, respectively.
